# Meta-Analysis and Gene Set Enrichment Relative to ER Status Reveal Elevated Activity of MYC and E2F in the “Basal” Breast Cancer Subgroup

**DOI:** 10.1371/journal.pone.0004710

**Published:** 2009-03-09

**Authors:** M. Chehani Alles, Margaret Gardiner-Garden, David J. Nott, Yixin Wang, John A. Foekens, Robert L. Sutherland, Elizabeth A. Musgrove, Christopher J. Ormandy

**Affiliations:** 1 Cancer Research Program, Garvan Institute of Medical Research, Sydney, Australia; 2 Department of Statistics and Applied Probability, National University of Singapore, Singapore, Singapore; 3 Veridex LLC, a Johnson & Johnson Company, North Raritan, New Jersey, United States of America; 4 Erasmus MC Rotterdam, Department of Medical Oncology, Josephine Nefkens Institute and Cancer Genomics Centre, Rotterdam, the Netherlands; Deutsches Krebsforschungszentrum, Germany

## Abstract

**Background:**

Breast cancers lacking the estrogen receptor (ER) can be distinguished from other breast cancers on the basis of poor prognosis, high grade, distinctive histopathology and unique molecular signatures. These features further distinguish estrogen receptor negative (ER−) tumor subtypes, but targeted therapy is currently limited to tumors over-expressing the ErbB2 receptor.

**Methodology/Principal Findings:**

To uncover the pathways against which future therapies could be developed we undertook a meta-analysis of gene expression from five large microarray datasets relative to ER status. A measure of association with ER status was calculated for every Affymetrix HG-U133A probe set and the pathways that distinguished ER− tumors were defined by testing for enrichment of biologically defined gene sets using Gene Set Enrichment Analysis (GSEA). As expected, the expression of the direct transcriptional targets of the ER was muted in ER− tumors, but the expression of genes indirectly regulated by estrogen was enhanced. We also observed enrichment of independent MYC- and E2F-driven transcriptional programs. We used a cell model of estrogen and MYC action to define the interaction between estrogen and MYC transcriptional activity in breast cancer. We found that the basal subgroup of ER− breast cancer showed a strong MYC transcriptional response that reproduced the indirect estrogen response seen in estrogen receptor positive (ER+) breast cancer cells.

**Conclusions/Significance:**

Increased transcriptional activity of MYC is a characteristic of basal breast cancers where it mimics a large part of an estrogen response in the absence of the ER, suggesting a mechanism by which these cancers achieve estrogen-independence and providing a potential therapeutic target for this poor prognosis sub group of breast cancer.

## Introduction

Breast cancers are routinely classified into estrogen receptor positive (ER+) and estrogen receptor negative (ER−). These tumor types have distinct molecular phenotypes [1]. ER+ cancers may respond to anti-estrogens such as tamoxifen [2], although a significant proportion demonstrate resistance to endocrine therapy. ER− tumors fail to respond to endocrine therapy and have a poor prognosis when compared to ER+ tumors [2]. The genetic pathways utilized by ER− tumors to proliferate in the absence of a mitogenic estrogen (E_2_) signal are poorly understood. Elucidation of these pathways is required for the development of improved therapies for ER− patients. Currently the only targeted therapy for ER− tumors is a monoclonal antibody against the ErbB2 receptor (ERBB2), Herceptin, which is indicated in ER−/Progesterone Receptor (PGR)−/ERBB2+ patients. The genetic mechanisms responsible for proliferation in ER− tumors could also allow ER+ tumors to exhibit intrinsic or acquired endocrine resistance and so develop a functional ER− tumor status.

Several studies have defined sets of genes with differential expression levels between ER+ and ER− tumor types [3]–[5]. Others have defined the smallest gene set that discriminates between molecular subtypes such as luminal (predominantly ER+), ERBB2+ and molecular basal (predominantly high grade/ER−/PGR−/ERBB2−/basal cytokeratin +) [1], with a view to producing better prognostic markers. These gene sets show a small overlap restricted to only the most differentially expressed genes [4], preventing the definition of common pathways. Integration of data from multiple studies by a meta-analysis provides the statistical power necessary to define common genetic pathways and to provide new biological insight into the cause of phenotypic diversity in breast cancer. A meta-analysis minimizes individual study biases, and identifies genes with small but consistent expression changes that might not have passed significance thresholds in individual studies.

We have conducted a meta-analysis of five independent breast cancer cohorts with the objective of producing a comprehensive measure of differential expression between ER+ and ER− tumors for every probe set on the Affymetrix HG-U133A chip. We present the first study with sufficient numbers of tumors to separate the confounding effects of grade and ER status. The genetic pathways and mechanisms active in ER+ and ER− tumors were elucidated using two different approaches to functional annotation analysis of the meta-analysis results – testing for over-representation of functional categories using Database for Annotation, Visualization, and Integrated Discovery (DAVID) [6], and for enrichment of public and in-house gene lists using gene set enrichment analysis (GSEA) [7]. We related the functional annotation and GSEA results to the subtypes of breast cancer in three independent validation datasets. We show that enhanced transcriptional activity of MYC within the basal subgroup of ER− breast cancer mimics aspects of the transcriptional response to estrogen seen in ER+ cancers. This finding provides a mechanism that allows ER− tumors to overcome the absence of ER and establishes MYC and its transcriptional targets as candidates for the development of novel therapies for the basal subgroup of breast cancer.

## Materials and Methods

### Data Collection

Five datasets of primary breast tumors profiled on Affymetrix HG-U133A microarrays [8]–[11] were used in this meta-analysis. Data from [11] were split into two datasets, those from Uppsala University Hospital (Sotiriou.Uppsala), and the others from the John Radcliffe Hospital who did not receive adjuvant systemic therapy (Sotiriou.JRH.Untreated). HG-U133B data from [9] were excluded. Each dataset was normalized and log2 probe-set intensities calculated using the Robust Multichip Averaging (RMA) algorithm [12]. Subset datasets of Elston-Ellis Grade 3 tumors containing a total of 82 ER− and 101 ER+ patients were then created for use in the meta-analysis (refer Text S1 for patient characteristics).

Three independent RMA-normalized breast cancer datasets [13]–[15] were used for validation of the meta-analysis and molecular subtype analysis (Text S1). The Richardson dataset [13] of Grade 3 tumors and normal samples, designated tumors as “Basal”, “BRCA1” (positive for *BRCA1* mutations) or “Non BLC” (non-basal-like cancer) by immunohistochemistry (IHC). For the Wang dataset [14] we used the relative transcript levels of *ER* (probe set ID 205225_at), *PGR* (208305_at), *ERBB2* (216836_s_at) and *KRT5* (201820_at) to identify basal samples. ER status was not available for the Pawitan cohort [15]. These samples had however been classified into the molecular subtypes of Perou *et al.* [16] by Pawitan *et al.* [15], (refer the GSE1456 series deposited in the Gene Expression Omnibus http://www.ncbi.nlm.nih.gov/geo), and these classifications were retained in our study.

### Meta-analysis

Meta-analysis [17] was carried out using functions implemented in the *GeneMeta* package [18]. The change in a gene's expression level between ER+ and ER− tumors in each individual study was expressed as an effect size, which is a unit-free standardized mean difference between conditions measuring the magnitude of a covariate effect corrected for sample size bias. The effect size of each HG-U133A probe set in each dataset was entered into a random effects model which takes into account intra- and inter-study variability to produce a Z score as described in Text S1. A negative Z score indicates a probe set with higher intensity in ER− tumors. The statistical significance of differential expression was calculated by converting the Z scores to P-values which were then adjusted for multiple testing using the Benjamini-Yekutieli (BY) correction [19]. The transformed weighted average ratio (tWAR), which provides an indication of the fold-change between ER+ and ER− tumors, was calculated as described in Text S1.

### Functional annotation analysis

Sets of selected genes were tested for over-representation of functional annotation categories, including gene ontology (GO) and protein domain categories, using tools within DAVID version 2007 [6] (refer Text S1 for category details). The BY correction for multiple testing was applied to the EASE scores, and the significance threshold set at adjusted P≤0.05. Cell cycle maps were obtained from the GenMapp database [20], and genes within the map colored using tWARs and adjusted P-values from the meta-analysis.

### Statistical analysis of validation datasets

Complete linkage hierarchical clustering was performed on data scaled so that all probe-sets shared the same mean and variance, using the euclidean distance metric in the *stats* package in R [21]. The difference in mean probe set intensities between sets of genes in basal and non-basal ER− samples, or between basal and normal samples in the validation data sets was assessed using a two-sided paired t-test. For individual genes of interest, the difference in mean intensity was assessed using a two-sided Welch two-sample t-test.

### Analysis of MYC and E_2_ datasets for differential expression

Transcript profiling data from the studies of Bild *et al.* [22], Carroll *et al.* [23] and the in-house study of Musgrove *et al.* [24] (GSE11791 series deposited in the Gene Expression Omnibus) were RMA-normalized and analyzed for differential expression using LIMMA [25]. No intensity or fold-change filters were used, and the significance threshold for differential expression was set at BY adjusted P<0.05.

### Gene Set Enrichment Analysis (GSEA)

GSEA [7] was used to determine if the members of a given gene set were generally associated with “ER−” tumor status, and was therefore performed on all 22,283 probe sets on the HG-U133A chip ranked by meta-analysis Z score from most negative to most positive. The gene list was collapsed to unique gene symbols using the default capabilities. The maximum gene set size was fixed at 1500 genes, and the minimum size fixed at 15 genes. 1000 random sample permutations were carried out, and the significance threshold set at FDR<0.05. If a gene set had a positive enrichment score, the majority of its members had higher expression in ER− tumors than in ER+ tumors, and the set was termed “enriched” in ER− tumors. If a set had a negative enrichment score it was termed “depleted” in ER− tumors. An initial screening of gene sets enriched in ER− tumors was carried out using the Molecular Signature Database (MolSigDB) c1.v2, c2.v2 and c3.v2 gene sets current as of March 2007 (data not shown). On consideration of the results, other published gene sets relating to the action of E_2_, MYC and E2Fs were curated and added to the MolSigDB lists in a second GSEA screen.

## Results

### Meta-analysis of ER+ vs. ER− tumors

A meta-analysis approach [17] was used to obtain overall measures for gene expression in ER+ and ER− tumors from 5 datasets. All datasets consisted of fresh-frozen primary breast cancers profiled on the Affymetrix HG-U133A platform with information on ER status and grade. The meta-analysis was restricted to Grade 3 tumors (82 ER−, 101 ER+), to overcome the association between grade and ER status, and was verified in three independent datasets. Details of the 8 data sets used are provided in Text S1. Measures for ER status association were obtained for all 22,283 HG-U133A probe sets and are presented in the searchable Table S1, of which 2141 (9.6%) were differentially expressed between ER+ and ER− tumors with adjusted P<0.01. This set is referred to as the ER status-associated (ERA) genes.

To explore whether the results from Grade 3 tumors may be extended to tumors of other grades, we performed a Principal Components Analysis of Grade 2 tumors using the ERA genes (Figure S1). The ERA genes separated the ER+ and ER− Grade 2 tumors indicating that many of the genes differentially expressed in Grade 3 tumors are also differentially expressed in the Grade 2 tumors.

**Figure 1:**
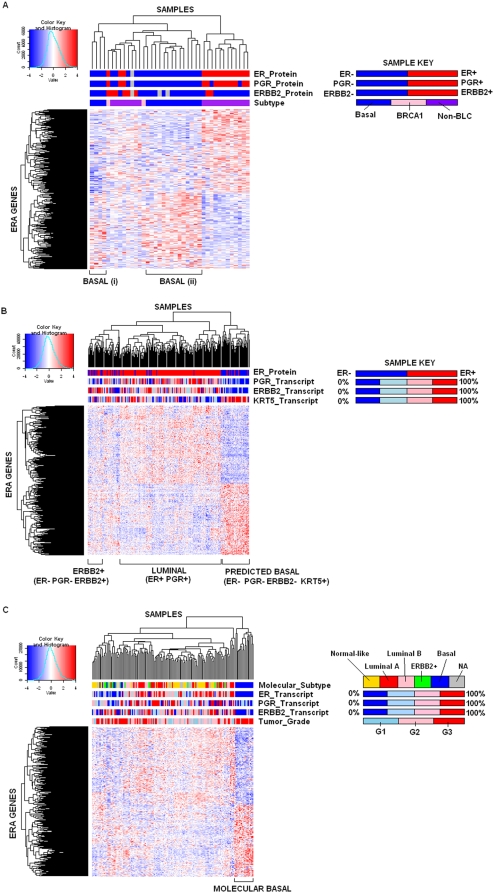
Hierarchical clustering of ERA genes in cancer samples from A. [13], B.[14], and C.[15]. Each row represents a gene; each column represents a sample. The expression level of each gene within a sample, relative to that gene's mean expression across all the samples, is indicated using a red-blue color scale with red indicating high expression. The dendrogram at the top indicates the similarities between the sample expression profiles while the side-dendrogram indicates the similarities between gene expression patterns. Figure 1A data is derived from the Richardson dataset [13] and the top color bars indicate the following: “ER_Protein” – ER status determined using IHC; “PGR_Protein” - PGR status determined using IHC; “ERBB2_Protein” - ERBB2 status determined using IHC; “Subtype” – Subtype determined from IHC results (Basal, *BRCA1* mutation positive, or non-basal-like carcinoma (“Non-BLC”)) (see sample key). Figure 1B data is derived from the Wang dataset [14] and the top color bars represent the following: “ER_Protein” – ER status as determined using ligand binding assay or IHC; “PGR_Transcript”, “ERBB2_Transcript” and “KRT5_Transcript”: relative expression measured from quantiles of probe set intensities as described in “Data Collection” in Materials and Methods (see sample key). Figure 1C data is derived from the Pawitan dataset [15] and the top color bars represent the following: “Molecular_Subtype” – determined by correlation to the normal-like, Luminal A, Luminal B, ERBB2+ and basal molecular subtypes [16]; “ER_Transcript”– Relative expression of the HG-U133A ESR1 transcript 205225_at measured in quantiles; “PGR_Transcript”, and “ERBB2_Transcript” as above; and “Tumor_Grade” – Elston Ellis grading (see sample key).

The ERA genes were used to cluster the tumors from three independent validation datasets, visualized in the form of heatmaps (Figure 1). In each heatmap, two main gene clusters were produced. The top cluster in each heatmap corresponds to ERA genes that are more highly expressed (red in color) in ER+ tumors and expressed at lower level (blue in color) in ER− tumors. The bottom cluster corresponds to ERA genes that show the converse pattern of expression. The ER status of the tumors is indicated along the top of the heatmap and demonstrates that the ERA gene set can correctly delineate ER+ and ER− tumors in the independent data sets, so validating their intended capacity. Importantly, within the ER− tumors in all three datasets, the ERA genes clearly delineated the basal (Richardson set, Figure 1A), predicted basal (Wang set, Figure 1B) or molecular basal (Pawitan set, Figure 1C) subtypes from the ER−/ERBB2+ subtypes. The expression of ERBB2 is indicated along the top of each heatmap, as is the expression of Keratin 5 (KRT5) in Figure 1B, and appropriate enrichment of these genes in the subtypes is clearly demonstrated. The clustering also distinguished the luminal subtype. Thus the genes that distinguish ER status in our 5 discovery datasets clearly operate in 3 independent datasets to achieve not only the separation of tumors by ER status but also by molecular subtype.

### ER− tumors show higher expression of proliferation genes than ER+ tumors of the same grade

**Figure 2:**
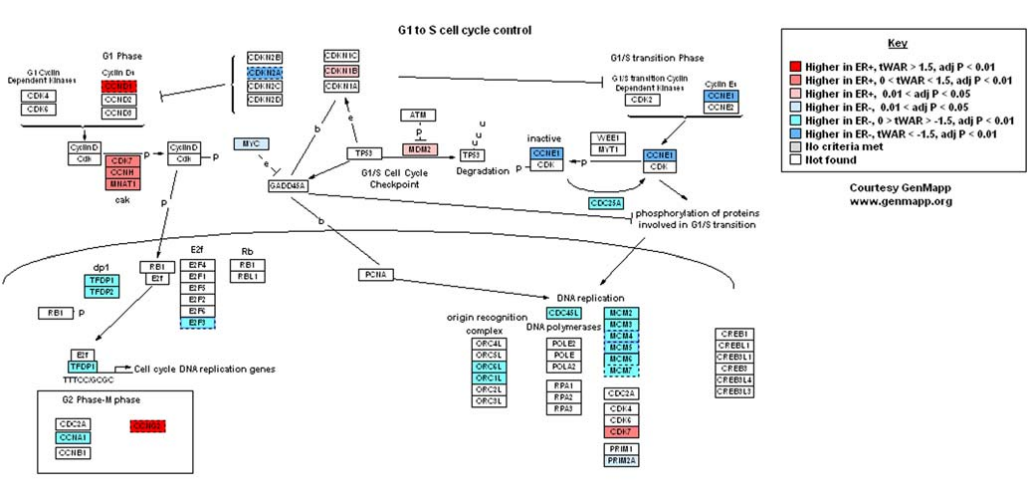
Meta-analysis results for genes in the GenMapp representation of “G_1_ to S-phase Cell Cycle Control”. Genes are colored by whether they have higher expression in ER+ or ER tumors from the meta-analysis, with the foldchange and significance of this over-expression represented by the transformed Weighted Average Ratio (tWAR) and BY-adjusted P-value (adj P) respectively.

The ERA genes were analyzed using the “DAVID” tools which test for over-representation of gene ontologies, pathways and protein domains. All 12 categories significantly over-represented in the ERA genes were related to the cell cycle and mitosis (Table 1). These cell-cycle related ERA genes showed predominantly higher expression in ER− tumors, showing that ER− tumors have a higher proliferation rate than ER+ tumors even when the tumors are of the same high grade (Table S2). This is illustrated by an overlay of the meta-analysis results on pathway maps of key cell cycle control genes, namely the “G1 to S Cell cycle control” (Figure 2) and “KEGG Cell Cycle” (Figure S2) maps in the GenMAPP database.

**Table 1:** Functional annotation categories from DAVID [6] significantly over-represented in ERA genes.

Category	Term	Count	Un-adjusted P	Adjusted P
SP_PIR_KEYWORDS	cell cycle	83	2.40E-12	8.87E-08
GOTERM_BP_ALL	cell cycle	142	1.35E-11	2.49E-07
KEGG_PATHWAY	HSA04110∶CELL CYCLE	41	4.55E-10	5.60E-06
GOTERM_BP_ALL	regulation of progression through cell cycle	94	1.10E-07	0.00092
GOTERM_BP_ALL	regulation of cell cycle	94	1.25E-07	0.00092
GOTERM_BP_ALL	mitotic cell cycle	50	7.34E-07	0.0045
GOTERM_BP_ALL	cell division	39	1.02E-05	0.038
GOTERM_BP_ALL	M phase	43	1.56E-05	0.047
GOTERM_BP_ALL	mitosis	36	1.66E-05	0.047
SP_PIR_KEYWORDS	cell division	35	1.92E-05	0.047
UP_SEQ_FEATURE	domain∶Kinesin-motor	13	2.27E-05	0.049
GOTERM_BP_ALL	M phase of mitotic cell cycle	36	2.37E-05	0.049

Many categories associated with cell cycle and cell division were significantly over-represented with BY adjusted P<0.05 in the comprehensive ERA gene list.

Within the ERA genes, cyclins A1, A2, B2, E1 and J, cyclin dependent kinase inhibitor 2A (*CDKN2A*) and CDK2 associated protein (*CDK2AP1*) show higher expression in ER− tumors whereas cyclins D1, G2 and H, *CDK7* and cyclin G-associated kinase (*GAK*) have higher expression in ER+ tumors. Several genes directly involved in DNA replication are more highly expressed in ER− tumors: for example, those encoding proteins in the origin-recognition complex (*ORC1L* and *ORC6L*), the minichromosome maintenance proteins *MCM2* to *MCM7*, and *CDC45L*. We clustered the genes in the cell cycle categories in the validation datasets, with the samples in forced order first by ER status and then by ERBB2 levels (Figure S3). The differential expression of proliferation associated genes was most pronounced in the basal subgroup of tumors, even when the tumors were of the same grade (Figure S3A), demonstrating the highly-proliferative nature of these tumors compared to other molecular sub types.

### Investigation of the meta-analysis results using GSEA

GSEA is a method that allows us to search the ERA gene set for transcript profiles indicative of underlying biological processes. An initial GSEA study was conducted testing the MolSigDB gene sets pertaining to chromosomal position, curated gene sets from publications, and conserved regulatory motifs [26] for enrichment in the ERA gene set. The complete list of significant associations is viewable by clicking the index.html file in Dataset S1 to launch your browser. Inspection of the results revealed the very prominent enrichment in ER− tumors of many gene sets relating to MYC and E2F activity. Although other themes emerge from this analysis we have concentrated our efforts on these findings. *MYC*, like cyclin D1, is a target of E_2_ and can rescue cell proliferation in anti-estrogen arrested MCF-7 cells [27]. We conducted a second GSEA screen after adding extra published and in-house gene sets (see Materials and Methods) relating to E_2_-, MYC- and E2F-activity in cell-lines, with the added datasets indicated by a prefix of “MCA” (Text S2).

Gene sets associated with ER+ status [4], [28] and good outcome in breast cancer [28] were depleted in ER− tumors (Text S2.A), and genes associated with ER− status [28] and poor outcome [28] were enriched in ER− tumors, showing that the meta-analysis results concur with those of single cohorts. Several gene sets associated with the cell cycle were enriched in ER− tumors, supporting the results from the functional annotation using DAVID (Text S2.B). The gene sets enriched or depleted with false discovery rate (FDR)<0.05 were examined more closely and assigned to biological themes; many sets were associated with chromosomal position, E_2_-action, MYC action and E2F-action. We report on these categories in detail in subsequent sections.

### Specific cytobands with expression differences between ER+ and ER− tumors

DNA copy number alteration shows regional differences between subtypes of ER+ and ER− tumors [29], [30]. Using GSEA, we identified cytoband loci containing sets of genes with expression differences between ER+ and ER− tumors (Table 2); genes in these cytobands are marked in the tables of ERA genes in Table S1. Enriched in ER− tumors were genes within 6p21, 7q32–q33, 10p13 and 21q22 which show gain by comparative genome hybridization (CGH) in basal tumors [31] and 1p34 which shows loss of heterozygosity in ER+ tumors [30]. We note that gain in 21q22 is associated with poor prognosis [31] and that this cytoband contains the cell cycle associated ERA genes *CHAF1B* and *S100B* that have elevated expression in ER− tumors. Depleted in ER− tumors were the cytobands 4p16, 5q11–13, 5q22, 5q31, 14q22–23 which show more frequent loss in basal tumors, and 16p11–p13 which shows more frequent gain in luminal tumors [30]. The cytobands 14q11–12, 16p12–13, 17q21 and 17q24 were also depleted in ER− tumors and low resolution CGH mapping suggests that 14q, 16p and 17q have increased loss in basal-like tumors [29]. Genes within 17q21 were generally depleted in ER− tumors, yet amplification of 17q12–21 is common in ERBB2+ tumors, which are usually ER−, and amplification of this region predicts a worse prognosis [31]. Our study delineated novel sites enriched in ER− tumors at the cytobands 2p13, 2p16, 2p21, 2p25, 3q25, 3q29, 6q16, 6q21, 9q21, 11q21–22, 12p13 and 22q13 and depleted in ER− tumors at 3p21, 11q13, and 12q13. These regions may represent previously undetected DNA copy number alterations or epigenetic silencing/reactivation events. ERA genes that had differential expression consistent with enriched/depleted cytobands and are involved in cell cycle and/or cell proliferation are marked in Table 2. Dysregulation of these genes may contribute to the differences in proliferation rates between ER+ and ER− cancers.

**Table 2:** Chromosomal position gene sets enriched or depleted in ER− tumors with FDR<0.05.

Gene Set Name	FDR	ERA genes relating to cell cycle and/or cell proliferation*	Enriched or depleted in ER− tumors
CHR1P34	0.0017	*NASP*, *CDC20*, *KIF2C*, *CDCA8*	Enriched
CHR2P13	0.010	*TGFA*	Enriched
CHR2P16	0.0081		Enriched
CHR2P21	0.0035	*MSH2*	Enriched
CHR2P23	0.044	*PPP1CB*	Enriched
CHR2P25	0.033		Enriched
CHR3P21	<0.0001	*APPL*, *CYB561D2*, *RBM5*, *TUSC2*, *TUSC4*	Depleted
CHR3Q25	0.022		Enriched
CHR3Q29	0.024		Enriched
CHR4P16	0.048	*GAK*	Depleted
CHR5Q11	0.0047		Depleted
CHR5Q12	0.048	*CDK7*	Depleted
CHR5Q13	0.00035	*CCNH*, *F2R*, *RAD17*, *RASA1*	Depleted
CHR5Q22	0.0094		Depleted
CHR5Q31	0.0050	*PURA*, *SKP1A*	Depleted
CHR6P21	0.036	*RNF8*, *PIM1*, *TUBB*, *GMNN*	Enriched
CHR6Q16	0.018		Enriched
CHR6Q21	0.0028	*HDAC2*	Enriched
CHR7Q32	0.0081		Enriched
CHR7Q33	0.041		Enriched
CHR9Q21	0.0044	*ANXA1*	Enriched
CHR10P13	0.0040		Enriched
CHR11Q13	0.0020	*CCND1*, *VEGFB*	Depleted
CHR11Q21	0.0058		Enriched
CHR11Q22	0.020		Enriched
CHR12P13	0.010	*NOL1*, *CDCA3*	Enriched
CHR12Q13	0.00076	*KRT18*, *MCRS1*	Depleted
CHR14Q11	0.00054		Depleted
CHR14Q12	0.050		Depleted
CHR14Q22	0.0097	*CGRRF1*, *HSPA2*	Depleted
CHR14Q23	0.021	*MNAT1*	Depleted
CHR16P11	0.022		Depleted
CHR16P12	0.0079		Depleted
CHR16P13	<0.0001	*E4F1*, *EMP2*, *GFER*, *UBE2I*	Depleted
CHR17Q21	0.0082	*HEXIM1*	Depleted
CHR17Q24	0.0039		Depleted
CHR21Q22	0.013	*CHAF1B*, *S100B*	Enriched
CHR22Q13	0.024	*MCM5*, *GTSE1*	Enriched

* Genes are in cell cycle categories in Table S2 and/or the GO0008283 cell proliferation ontology.

### Direct transcriptional targets of ER are significantly depleted in ER− tumors, but indirect E_2_-induced target genes are enriched in ER− tumors

We expected that much of the gene expression differences between ER+ and ER− tumors might be due to the ability of ER+ tumors to respond to E_2_. To distinguish the direct and indirect effects of E_2_ we added datasets of direct ER targets derived by chromatin immunoprecipitation based studies, and datasets containing both direct and indirect targets characterized by early response to E_2_ (Text S2.C, with genes within these datasets marked in Table S1). Direct ER targets were significantly depleted in ER− tumors (8 sets FDR<0.0001 to 0.023, 1 set = 0.052), with depletion of both E_2_-induced and E_2_-repressed direct targets [23] (FDR 0.0013 to 0.021). Surprisingly, sets containing indirectly E_2_-induced genes were significantly enriched in ER− tumors (Text S2.C) including sets from [24](FDR = 0.015) and [32] (FDR = 0.0047), while several sets of genes down-regulated in response to E_2_ remained significantly depleted in ER− tumors (Text S2.C). An explanation of these observations may be that transcriptional regulators within the E_2_ pathway may become activated independently of the ER in ER− tumors, driving the transcription of indirect targets of E_2_.

### High expression of MYC-induced genes and concomitant low expression of MYC-repressed genes in the basal subgroup

As seen in Table 3 and Text S2.D, several sets of direct targets of MYC derived from various experimental and tumor systems, genes containing MYC binding motifs and MYC-induced genes, were enriched in ER− tumors. Prominent among these were sets of genes induced by MYC in human primary mammary epithelial cell cultures (HMECs) [22] and in MCF-7 breast cancer cells [24](both with FDR<0.0001). Notably, the subset of MYC-induced genes in MCF-7 cells that was also induced by E_2_ [24] was highly enriched in ER− tumors (FDR<0.0001) and the enrichment plot for this gene set is shown in Text S2.D.i. The enrichment of the MYC-induced genes in MCF-7 cells taken as a whole (FDR<0.0001) was greater than the enrichment of E_2_-induced genes from the same study (FDR = 0.015). MYC-repressed genes in MCF-7 cells taken as a whole were depleted in ER− tumors (FDR<0.0001) (Table 4).

**Table 3:** Details of MYC- and E2F-related sets enriched in ER− tumors with FDR<0.0001.

Gene Set Name	Reference	Experimental System	Description
MCA.Baliciunate_p130.G1_B	[37]	MEFs (G_1_ phase), ChIP	P130_B
MCA.Musgrove_E2_U and Myc_U	[24]	Anti-estrogen arrested MCF-7.	E2_U and MYC_U
		6 hours after E_2_-induction, or	
		6 hours after MYC- induction.	
MCA.Baliciunate_E2F4.G0_B	[37]	MEFs (G_0_ phase), ChIP	E2F4_B
MCA.Musgrove_Myc_U	[24]	Anti-estrogen arrested MCF-7.	MYC_U
		6 hours after MYC- induction.	
MCA.Baliciunate_p107.G1_B	[37]	MEFs (G_1_ phase), ChIP	P107_B
MCA.BLACK.2005.TOP.100.E2F1-INDUCED GENES.SUPPLTABLE2	[36]	MEFs	E2F1_U
VERNELL_PRB_CLSTR1	[35]	U2OS	E2F_U (Up-regulated by E2Fs 1,2 or 3 and down-regulated by pRB and p16)
MCA.Baliciunate_p130.G0_B	[37]	MEFs (G_0_ phase), ChIP	P130_B
MCA.Baliciunate_E2F4.G1_B	[37]	MEFs (G_1_ phase), ChIP	E2F4_B
YU_CMYC_UP	[54]	Non-transgenic murine model for B-cell lymphoma.	MYC_U
MCA.Bild_Myc.LIMMA_U	[22]	HMEC	MYC_U
SGCGSSAAA_V$E2F1DP2_01	[26]	NA	E2F1_M and TFDP2_M
REN_E2F1_TARGETS	[38]	WI-38 Primary human fibroblasts, ChIP	E2F1_B and E2F4_B
MCA.Zeller_Myc_U and Myc_B	[33]	Human B lymphoid tumor, ChIP coupled with pair-end ditag sequencing analysis (ChIP-PET)	MYC_U and MYC_B
MCA.BLACK.2005.TOP.100.E2F3-INDUCED GENES.SUPPLTABLE3	[36]	MEFs	E2F3_U
V$E2F1_Q6	[26]	NA	E2F1 _M
V$E2F4DP2_01	[26]	NA	E2F4_M +TFDP2_M
V$E2F1_Q6_01	[26]	NA	E2F1 _M

Tables are sorted by increasing FDR. The suffixes “U”, “D”, “B” and “M” refer to whether the genes in the set are **U**p-regulated or **D**own-regulated by the molecule in question, **B**inding Partners (or direct targets) of the molecule identified via chromatin immunoprecipitation (ChIP), or contain Binding **M**otifs for the molecule in their promoter regions.

**Table 4:** Details of MYC-related sets depleted in ER− tumors with FDR<0.0001.

Gene Set Name	Reference	Experimental System	Description
MCA.Musgrove_ Myc_D	[24]	Anti-estrogen arrested MCF-7.	MYC_D
		6 hours after MYC-induction.	
MCA.Musgrove_E2_D and Myc_D	[24]	Anti-estrogen arrested MCF-7.	E2_D and MYC_D
		6 hours after E_2_-induction, or	
		6 hours after MYC-induction.	

Tables are sorted by increasing FDR. The suffix “D” refers to whether the genes in the set are **D**own-regulated by the molecule in question.

**Figure 3:**
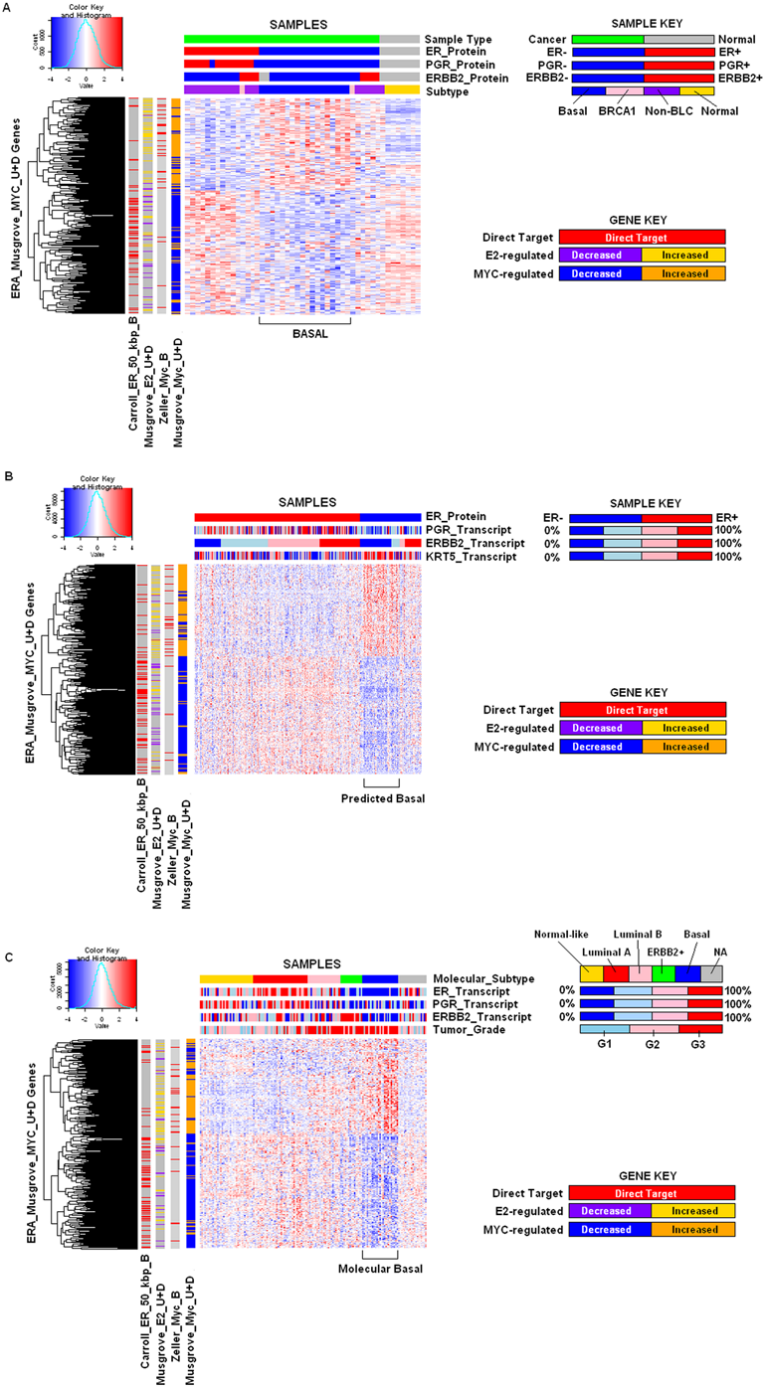
Hierarchical clustering of 500 MYC-responsive ERA genes [24] in validation datasets from A.[13], B.[14], and C.[15]. The first top color bar of Figure 3A. represents whether the sample is a breast cancer or from normal breast tissue. The remaining top color bars of Figure 3A are equivalent to those in Figure 1A. The colors in the side color bars represent the following: yellow = an E_2_-induced gene, purple = an E_2_-repressed gene, orange = a MYC-induced gene, blue = a MYC-repressed gene and red indicates a gene which is a direct target of ER (“ER_B” suffix), or a direct target of MYC (“Myc_B”). Moving from right to left, for each gene, the first side color bar represents the transcriptional response to MYC in MCF-7 cells [24] (Musgrove_Myc_U+D). The next color bar represents whether the gene was classified as being a direct target of MYC in B cell lymphomas [33] (Zeller_Myc_B). The next two color bars represent how this gene was regulated by E_2_ in MCF-7 cells [24] (Musgrove_E2_U+D), and whether it contained an ER-binding site with 50 kbp of the promoter region [23] (Carroll_ER_50 kbp_B). The top color bars of Figure 3B are equivalent to those of Figure 3B, and the side color bars are the counterparts of those in Figure 3A. The top color bars of Figure 3C. are equivalent to those of Figure 3C, and the side color bars are the counterparts of those in Figure 3A.

In the ERA genes, 84% of those that were MYC-induced in MCF-7 cells had significantly higher expression in ER− tumors, and 87% that were MYC-repressed had lower expression in ER− tumors (Table S1). We therefore examined, in the three validation datasets, the expression of the ERA genes that were regulated by MYC in MCF7 cells (Figure 3). With the samples forced to order first by ER status and then by ERBB2 level we examined the differential patterns of expression of the Musgrove_Myc_U+D subset of the ERA genes relative to molecular subtype and the ability of this gene set to distinguish directly- and indirectly-regulated E_2_ and MYC targets. In the left hand side color bars of the heatmaps we have marked the direction of MYC regulation in MCF7 cells (Musgrove_Myc_U+D) [24] as well as the direct targets of MYC defined in B cell lymphoma (Zeller_Myc_B) [33]. Consistent with the results of the meta-analysis and GSEA, the MYC-induced genes were generally higher in ER− tumors and the MYC repressed genes were lower in ER− tumors in all three cohorts. The MYC direct targets followed the same trends as the MYC-regulated genes as a whole.

Since MYC is a direct target of E_2_, we determined which of these genes are direct targets of ER (Carroll_ER_B) [23], and how they are regulated by E_2_ in MCF7 cells (Musgrove_E2_U+D) [24]. As shown by the left hand side color bars, and consistent with the GSEA results, the direct targets of ER had higher expression in ER+ tumors in the three validation datasets. Also as expected, many of the MYC-regulated genes overlapped with E_2_-regulated genes from the same experiment. The overlapping ERA E_2_-induced and MYC-induced genes had higher expression in ER− tumors, and the overlapping ERA E_2_-repressed and MYC-repressed genes had lower expression in ER− tumors.

Importantly, the higher expression of MYC-induced ERA genes was more pronounced in the basal subgroup than in ER− tumors with high ERBB2 expression (P<0.0001 for all three datasets), (Figure 3). Similarly, the lower expression of MYC-repressed ERA genes was generally more pronounced in the basal subgroup than in the ER−/ERBB2+ samples (P<0.0001 for all three datasets), (Figure 3). When ERA genes regulated by MYC in ER− HMECs [22] were used to cluster the validation datasets (Figure S4), we observed very similar results with respect to the basal subtype, demonstrating that the MYC pathway is more active in the basal subgroup and that it mimics estrogen action.

### Genes with E2F binding motifs and direct targets of the E2F family are enriched in ER− tumors

The E2F family and the proteins that modulate E2F activity are important for cell cycle progression (reviewed in [34]) In the meta-analysis, all probe sets for *E2F3* and *E2F8* showed significantly higher intensity in ER− tumors (adjusted P<0.0001 and adjusted P = 0.034 respectively) (Text S3). All probe sets for *E2F1* and *E2F5* and one probe set for *E2F4* had higher intensity in ER− tumors (un-adjusted P<0.05), but did not pass the adjusted P significance threshold. Probe sets for *TFDP1* and *TFDP2*, members of the DP transcription factor family, whose products form heterodimers with E2Fs 1 to 6 producing transcription factor complexes that bind to DNA [34], were significantly higher in ER− tumors (*TFDP1*, adjusted P = 0.0073; *TFDP2*, adjusted P = 0.0023). One probe set for *p130*, a pocket protein that binds principally to E2Fs 4 and 5 converting them into repressor complexes, had significantly lower intensity in ER− tumors (adjusted P = 0.0053) with the other probe set showing the same trend. For each gene above, the tWAR, indicating fold-change, was small, with the possible exception of the two probe sets for *E2F3* (tWAR = 1.48-fold and 1.88-fold).

In the GSEA screen, several gene sets significantly enriched in ER− tumors were associated with E2F activity (Table 3 and Text S2.E). Nineteen sets contained genes with conserved DNA binding motifs for E2F family members, particularly E2F1 and E2F4 [26]. Different members of the E2F family can bind to the same genes depending on the conditions such as the phase of the cell cycle, and these motif sets had many genes in common. Supporting the observations for genes with conserved E2F motifs, a MolSigDB list of genes up-regulated by E2Fs 1, 2 or 3 and down-regulated by pRB and p16INK4A in U2OS cells [35] was enriched in ER− tumors (FDR<0.0001) (Table 3). The complementary set of genes down-regulated by E2Fs 1, 2 or 3 and up-regulated by pRB and p16INK4A [35] was depleted (FDR = 0.038).

In order to better understand the roles E2Fs might play in the regulation of proliferation in ER− tumors, we included additional published sets of direct targets of E2Fs 1, 4 and 6, as well as sets of genes regulated by E2F1 or E2F3 (Text S2.E). A list of genes regulated by E2F1 in mouse embryonic fibroblasts (MEFs) was enriched with FDR<0.0001 (Table 3), as was a set of genes regulated by E2F3 from the same study [36]. Several sets of direct targets of E2F1 and E2F4 were significantly enriched in ER− tumors (Table 3 and Text S2.E). The sets enriched with FDR<0.0001 included direct targets of E2F4 in MEFs during either G_0_ [37] or early G_1_ stage of the cell cycle [37], genes bound by both E2F4 and E2F1 in WI-38 primary human fibroblast cells [38], and genes bound by E2F4 and not E2F1 in the same study [38]. The pocket proteins p130 and p107 bind to E2F4 converting it to a repressor complex, and consistent with this association, direct targets of p130 in MEFs during G_0_ or G_1_ [37] were enriched with FDR<0.0001, as were targets of p107 during G_1_ [37].

To examine if the enrichment of E2F activity differed between the subtypes of ER− tumors, we clustered the validation tumor datasets using ERA genes that were associated with E2F activity (Figure S5). We confirmed that the enriched set of direct targets of E2F4 in MEFs had higher expression in samples with low levels of ER. These genes had particularly high expression in the basal subgroup compared to the ER−/ERBB2+ subgroup in all three datasets (all with P<0.0001), (Figure S5). The ERA genes overlapping with sets of genes activated by E2Fs 1, 2 or 3 in U2OS cells [35], by E2F1 or E2F3 in MEFs [36] and all the enriched gene sets of direct targets of E2Fs 1, 4 or 6 also showed differential expression in the basal tumors (data not shown). ERA genes that were direct targets of E2F4 and over-expressed in basal ER− tumors were also over-expressed with respect to normal tissue (P<0.0001), (Figure S5A).

To ascertain if the enrichment noted for the MYC and E2F sets was entirely due to the presence of proliferation-associated genes in these sets, we removed genes in the GO “cell cycle” (GO∶0007049) and “cell proliferation” (GO∶0008283) categories from the relevant sets enriched with FDR<0.0001. GSEA showed that these “proliferation depleted” sets remained as highly enriched as their complete counterparts, and the leading edges of the “proliferation depleted” MYC and E2F sets shared few common genes (data not shown). These results indicate that outside the shared proliferation-associated genes, MYC-action and E2F-action are separate forces contributing independently to the transcriptional differences between ER− and ER+ tumors.

We also found that the cytobands that were differentially expressed between ER+ and ER− tumors did not contain MYC or genes known to influence MYC-activity (such as the binding partners MAD or MAX). Similarly, the enriched or depleted cytobands did not contain any members of the E2F family that were differentially expressed in the meta-analysis, or any genes known to influence E2F-activity (such as members of the families of DP transcription factors or pocket proteins). It is therefore unlikely that genetic alterations in these regions are the cause of the increased MYC and E2F activity in ER− tumors.

## Discussion

This study uses a novel meta-analysis approach to identify genes and genetic pathways associated with ER status in breast cancer. Importantly, we restricted our analysis to grade 3 tumors because ER− tumors are almost exclusively of higher grade while ER+ tumors show greater diversity. Thus previously published lists of ER status-associated genes [3]–[5] may contain genes related to grade in addition to ER status. Functional annotation analysis of the 2141 ERA genes using DAVID and GSEA showed that categories associated with cell cycle were enriched in genes up-regulated in ER− tumors compared to ER+ tumors indicating that even at the same grade, ER− tumors exhibit a greater proliferation signal. Hierarchical clustering of the validation datasets revealed that cell-cycle associated genes are more highly expressed in the basal subgroup than in other ER− tumors. While other transcript profiling studies have reported that cell cycle and cell proliferation categories are over-represented in molecular basal tumors [39], they were unable to uncouple the effects of grade and ER status as we have done, due to restricted numbers of samples in their studies. Our results concur with those of a recent histopathological study confined to Grade 3 invasive ductal carcinomas, which found that the basal phenotype was highly significantly associated with high total mitotic count, a marker of increased proliferation [40].

In the GSEA screen, independent lists of direct targets of the E_2_/ER complex were depleted in ER− tumors as one would expect. The genes induced by E_2_ in MCF-7 cells in two studies were enriched in ER− tumors, and E_2_-repressed genes from four studies were depleted in ER− tumors. Consistent with our results, previous studies aiming to identify E_2_-induced genes over-expressed in ER+ tumors found fewer genes than expected [4], [41], and small subsets of E_2_-induced genes were observed to be over-expressed in ER− tumors [41], [42]. These apparent discrepancies were attributed to differences between tumors and cell lines [4], [41]. Our results provide an alternative explanation: aberrant activation of the E_2_-target MYC leads to a robust induction of a subset of genes characteristic of an E_2_ response. Consistent with this hypothesis, MYC is capable of rescuing cell cycle progression in MCF-7 cells arrested in G_1_ phase by pre-treatment with an estrogen antagonist [27] and a large proportion of the ERA genes regulated by E_2_ in MCF-7 cells are also MYC-regulated (Figure 3, Figure S4 and associated references). Unlike the direct targets of E_2_, both the direct targets of MYC in B-cell lymphoma and genes containing MYC binding motifs were enriched in ER− tumors. Our results support and extend those of Creighton *et al.* [32] who reported that a single list of genes which showed early and sustained induction by E_2_ in ER+ cell lines (labeled as “MCA.Creighton.Cluster B Genes_E2_Early.Sustained_U” in our GSEA screen) was enriched in ER− tumors in a cohort of Grade 1 to 3 tumors.

A wide range of *MYC* transcript levels by RT-PCR has been detected in both ER+ and ER− breast cancers [43]. In our meta-analysis, the expression of *MYC* was significantly higher in ER− tumors (adjusted P = 0.024), corroborating the results of a single cohort study [44]. *MYC* copy number amplification is associated with loss of ER and PGR in one cohort [45] and is not associated with ER positive status in another cohort [46]. Amplification of the *MYC* gene and/or over-expression of MYC protein is associated with high grade in some cohorts [44], [47]. Rhodes *et al.* [48] reported the presence of a set of genes activated by MYC in HMECs [22] in signatures of Grade 3 breast cancer.

We observed higher levels of *MYC* transcripts in the basal subtype compared to ER−/ERBB2+ samples in two of our validation cohorts. *MYC* is significantly amplified in tumors with *BRCA1* mutations which have a profile similar to basal tumors [49] but amplification of *MYC* is not correlated with the basal phenotype [46], suggesting that the high transcript levels of *MYC* we observed in ER− tumors may be due to factors other than *MYC* amplification. By classifying samples in the validation cohorts into the predicted subtypes of breast cancer, we made the novel observation that the elevated expression of MYC-induced genes, including MYC direct targets, and the lower expression of MYC-repressed genes distinguishes the basal subgroup of ER− tumors. Furthermore, in a concurrent study from this laboratory, we have also observed that expression of c-Myc with a predominant cytoplasmic staining pattern on IHC significantly correlates with the basal phenotype as determined by an ER−/PGR−/ERBB2−/KRT5/6+ staining pattern (McNeil CM, Musgrove EA and Sutherland RL, manuscript in preparation). The *MYC* transcript was over-expressed in the basal subtype compared to ER−/ERBB2+ samples in two of our validation cohorts, Richardson P = 0.036, Pawitan P = 0.00013, but not in Wang P = 0.964. *MYC* over-expression in basal cancer in other datasets can be distinguished on the basis of frequency [50], but our analysis of the five meta-analysis datasets indicates that it is not a prominent feature, ranking below the top 1000 differentially expressed mRNAs. Thus the strong transcriptional effect of MYC that we have detected suggests that more than altered MYC expression is contributing to its activity in basal breast cancer.

Our study also highlights the role of the E2F family in ER− tumors, and shows that their dysregulation may play a role in the proliferation of basal tumors. E2Fs are known to control the expression of genes important for cell cycle progression as well as genes involved in apoptosis and differentiation [34]. E2F binding motif gene sets were enriched, as were several lists of direct targets for E2F1, 4 and/or 6. As E2F family members overlap considerably in their binding specificity, we conclude that the direct binding of one or more members of the E2F family is increased in ER− tumors, but cannot specify which family member. The actions of the E2F family members are closely linked with those of MYC. The MYC protein can regulate the E2Fs [51], and vice versa [52]. Many of the genes regulated by various E2Fs during the early events of the cell cycle are also regulated by MYC [33], [52]. The *MYC* gene is bound by E2F4 but not p130 or p107 during G_0_ (but not G_1_) in MEFs [37]. MYC is a direct target of E2F1 in human fibroblasts [38]. *E2F3*, *E2F5* and *TFDP1*, which have higher expression in ER− tumors, are all induced by MYC in MCF-7 cells [24]. Zeller *et al.* [33] found the E2F1 binding motif is enriched 16-fold in clusters of MYC-binding genes, and 37-fold within the subset of E-box containing genes. In our study, the genes shared between gene sets relating to MYC- and E2F-action were predominantly proliferation-associated genes. In this context, we believe that dysregulation or constitutive activation of processes regulated by MYC in conjunction with increased E2F activity may lead to uncontrolled cell cycle progression and proliferation, such as we see in ER− tumors and particularly in the basal subtype within the ER− tumors. Kreike *et al.* [53] showed that the basal subtype may be further divided into five subgroups on the basis of gene expression profiles. A group of proliferation-associated genes was among those contributing to the clustering. The enrichment of MYC- and E2F-regulated genes in the genes partitioning the basal tumors in the cohort of Kreike *et al.* [53] may prove a valuable avenue of investigation. Our findings may also have important therapeutic implications. A recent bioinformatic study indicated a significant association between the set of genes activated by MYC in HMECs [22], and genes repressed on treatment of MCF-7 or HL-60 cells with the PI3K signaling pathway inhibitors wortmannin or LY-294002 [48]. MYC-activated genes had lower expression in cells treated with wortmannin or LY-294002, suggesting that PI3K inhibitors repress MYC-activation [48]. Our study provides strong evidence for MYC-action in basal tumors, suggesting that PI3K inhibitors, and other potential repressors of MYC-action, should be investigated as therapeutic candidates for these tumors which have no targeted therapies at present. We anticipate that the biological insights generated by this study will prove valuable in the development of new therapeutic strategies for the poor prognosis ER− tumors and in particular the basal subtype. We conclude that over-expression or constitutive activation of MYC, possibly in conjunction with elevated E2F activity, may contribute to increased proliferation in ER− breast tumors, particularly in the basal subgroup.

## Supporting Information

Dataset S1 GSEA results from first screen. The file contains a zipped archive of the HTML results of the second GSEA screen. Reports for enriched or depleted datasets may be accessed from the “index.html” link within the my_analysis.GseaPreranked.1219975950625.rpt.zip ZIP archive. Enrichment plots are available for each of the 50 most enriched and depleted gene sets.
(13.32 MB ZIP)

Figure S1 Principal components analyses (PCAs) using ERA genes on Grade 3 and Grade 2 ER+ and ER− samples. The ERA genes were identified by a meta-analysis of Grade 3 samples in five datasets (Farmer, Sotiriou.JRH.Untreated, Miller, Minn and Sotiriou.Uppsala). To demonstrate that these ERA genes also separate Grade 2 ER+ and ER− tumors, PCAs using the ERA genes were performed on Grade 2 samples from those same five datasets. The PCAs of Grade 3 samples in the Farmer, Sotiriou.JRH.Untreated, Miller, Minn and Sotiriou.Uppsala datasets are found in Figure S1A.i–v; PCAs of Grade 2 samples in the Farmer, Sotiriou.JRH.Untreated, Miller, Minn and Sotiriou.Uppsala datasets are found in Figure S1B.i–v.
(0.54 MB TIF)

Figure S2 Meta-analysis results for genes in the GenMapp representation of KEGG Cell Cycle. Genes are colored by whether they have higher expression in ER+ or ER tumors from the meta-analysis, with the foldchange and significance of this over-expression represented by the transformed Weighted Average Ratio (tWAR) and BY-adjusted P-value (adj P) respectively.
(0.08 MB TIF)

Figure S3 Hierarchical clustering of expression data from cell-cycle-associated genes in datasets from A. Richardson et al. (2006), B. Wang et al. (2005), and C. Pawitan et al. (2005). The two-way clustering of the ERA genes and samples previously (Figure 1) had indicated that the ERA genes were differentially expressed between the ER+, basal and ER−/ERBB2+ tumor subtypes. In order to clarify the behavior of the cell-cycle-associated genes in the different subtypes, we ordered the samples in the validation datasets primarily by ER status and secondarily by ERBB2 status, and then clustered only the cell-cycle-associated genes while maintaining the order of the samples. Figure S3A data is derived from the Richardson dataset (Richardson et al., 2006) and the top color bars indicate the following: “Sample Type” - whether the sample is a breast cancer or from normal breast tissue; “ER_Protein” - ER status determined using IHC; “PGR_Protein” - PGR status determined using IHC; “ERBB2_Protein” - ERBB2 status determined using IHC; “Subtype” - Subtype determined from IHC results (Basal, BRCA1 mutation positive, non-basal-like carcinoma (“Non-BLC”), or Normal tissue) (see sample key). Figure S3B. data is derived from the Wang dataset (Wang et al. 2005) and the top color bars represent the following: “ER_Protein” - ER status as determined using ligand binding assay or IHC; “ER_Transcript”, “PGR_Transcript”, “ERBB2_Transcript” and “KRT5_Transcript”: relative expression measured from quantiles of probe set intensities as described in “Data Collection” in Materials and Methods (see sample key). Figure S3C. data is derived from the Pawitan dataset (Pawitan et al. 2005) and the top color bars represent the following: “Molecular_Subtype” - determined by correlation to the normal-like, Luminal A, Luminal B, ERBB2+ and basal molecular subtypes (Sorlie et al. 2001); “ER_Transcript”, “PGR_Transcript”, and “ERBB2_Transcript” as above; and “Tumor_Grade” - Elston Ellis grading (see sample key). Two major clusters of cell-cycle-associated genes were observed in all three heatmaps; in each dataset, the highest expression of cell-cycle genes was observed in the basal samples, as marked.
(0.36 MB TIF)

Figure S4 Hierarchical clustering of expression data from 130 MYC-responsive ERA genes (Bild et al., 2006) in datasets from A. Richardson et al. (2006), B. Wang et al. (2005), and C. Pawitan et al. (2005). The ERA Bild_MYC_U+D genes are ERA genes that were also regulated by MYC in HMECs (Bild et al. 2006). We clustered these genes in the three validation datasets while maintaining the sample order. In Figure S4A, the top color bars are equivalent to those of Figure S3A. The colors in the side color bars represent the following: yellow = an E2-induced gene, purple = an E2-repressed gene, orange = a MYC-induced gene, blue = a MYC-repressed gene and red indicates a gene which is a direct target of ER (“ER_B” suffix), or a direct target of MYC (“Myc_B”). Moving from right to left, for each gene, the first two side color bars represent the transcriptional response to MYC in HMECs (Bild et al., 2006) (Bild_MYC_U+D), and in MCF-7 cells (Musgrove et al., 2008; McNeil et al., 2006) (Musgrove_Myc_U+D). The next color bar represents whether the gene was classified as being a direct target of MYC in B cell lymphomas (Zeller et al., 2006) (Zeller_Myc_B). The next three color bars represent how this gene was regulated by E2 in MCF-7 cells at 6 hours (Musgrove et al., 2008; McNeil et al., 2006) (Musgrove_E2_U+D), and at 3 hours (Carroll et al., 2006) (Carroll_3 hr_E2_U+D), and whether it contained an ER-binding site with 50 kbp of the promoter region (Carroll et al., 2006) (Carroll_ER_50 kbp_B). The top color bars of Figure S4B are equivalent to those of Figure S3B, and the side color bars are the counterparts of those in Figure S4A. The top color bars of Figure S4C are equivalent to those of Figure S3C, and the side color bars are the counterparts of those in Figure S4A. It can be seen that the majority of ERA MYC-induced genes have higher expression in the basal tumors, and the majority of ERA MYC-repressed genes have lower expression in basal tumors.
(0.48 MB TIF)

Figure S5 Hierarchical clustering of MCA.Baliciunate_E2F4.G0_B ERA genes (Baliciunate et al., 2005) in datasets from A. Richardson et al. (2006), B. Wang et al. (2005), and C. Pawitan et al. (2005). The MCA.Baliciunate_E2F4.G0_B genes are direct targets of E2F4 in mouse embryonic fibroblasts (Baliciunate et al., 2005). In Figure S5, the top color bars are equivalent of Figure S5A, S5B and S5C are equivalent to those of to those of Figure S3A, S3B and S3C respectively.
(0.35 MB TIF)

Table S1 The HG-U133A probe sets and their biological associations. A zipped EXCEL spreadsheet containing all 22,283 HG-U133A probe sets, along with Probe Set ID, Representative public database IDs (RefSeq transcript, Entrez, and Unigene), Gene Name, Gene Symbol, Z score, BY-adjusted P-value, status as an ERA gene, transformed Weighted Average Ratio, as well as whether they have an association with, E2-action, MYC-action, E2F-action, the cell cycle or significantly enriched or depleted chromosomal position in several different datasets. A negative “Overall Z score” indicates a probe set with higher intensity in ER− tumors.
(3.78 MB ZIP)

Table S2 Cell cycle genes over-expressed in ER− or ER+ tumors. Cell cycle associated genes, and their over-expression in ER− tumors or ER+ tumors from the meta-analysis.
(0.02 MB PDF)

Text S1 Supplementary Methods
(0.10 MB DOC)

Text S2 Behavior of major biological categories enriched or depleted in ER− tumors in GSEA. Tables are sorted by increasing FDR. Gene sets linked with Estrogen (E2), MYC or E2F activity have been labeled with suffixes “U”, “D”, “B”, “Dir” and “M” referring to whether the genes in the set are Up-regulated or Down-regulated by the molecule in question, Binding Partners (or direct targets) of the molecule identified via ChIP, other Direct targets of the molecule identified by the use of cycloheximide, or whether they contain conserved Binding Motifs for the molecule in their promoter regions.
(0.47 MB DOC)

Text S3 Behavior of probe sets for MYC, E2F and associated genes in the meta-analysis.
(0.09 MB DOC)

## References

[1] Sims AH, Howell A, Howell SJ, Clarke RB (2007). Origins of breast cancer subtypes and therapeutic implications.. Nat Clin Pract Oncol.

[2] Early Breast Cancer Trialists' Collaborative Group (2005). Effects of chemotherapy and hormonal therapy for early breast cancer on recurrence and 15-year survival: an overview of the randomised trials.. Lancet.

[3] Gruvberger SK, Ringner M, Chen Y, Panavally S, Saal LH (2001). Estrogen receptor status in breast cancer is associated with remarkably distinct gene expression patterns.. Cancer Res.

[4] Abba MC, Hu Y, Sun H, Drake JA, Gaddis S (2005). Gene expression signature of estrogen receptor alpha status in breast cancer.. BMC Genomics.

[5] Schneider J, Ruschhaupt M, Buness A, Asslaber M, Regitnig P (2006). Identification and meta-analysis of a small gene expression signature for the diagnosis of estrogen receptor status in invasive ductal breast cancer.. International Journal of Cancer.

[6] Dennis G, Sherman BT, Hosack DA, Yang J, Gao W (2003). DAVID: Database for Annotation, Visualization and Integrated Discovery.. Genome Biology.

[7] Subramanian A, Tamayo P, Mootha VK, Mukherjee S, Ebert BL (2005). Gene set enrichment analysis: A knowledge-based approach for interpreting genome-wide expression profiles.. PNAS.

[8] Farmer P, Bonnefoi H, Becette V, Tubiana-Hulin M, Fumoleau P (2005). Identification of molecular apocrine breast tumours by microarray analysis.. Oncogene.

[9] Miller LD, Smeds J, George J, Vega VB, Vergara L (2005). An expression signature for p53 status in human breast cancer predicts mutation status, transcriptional effects, and patient survival.. Proc Natl Acad Sci U S A.

[10] Minn AJ, Gupta GP, Siegel PM, Bos PD, Shu W (2005). Genes that mediate breast cancer metastasis to lung.. Nature.

[11] Sotiriou C, Wirapati P, Loi S, Harris A, Fox S (2006). Gene expression profiling in breast cancer: understanding the molecular basis of histologic grade to improve prognosis.. J Natl Cancer Inst.

[12] Bolstad BM, Irizarry RA, Astrand M, Speed TP (2003). A comparison of normalization methods for high density oligonucleotide array data based on variance and bias.. Bioinformatics.

[13] Richardson AL, Wang ZC, De Nicolo A, Lu X, Brown M (2006). X chromosomal abnormalities in basal-like human breast cancer.. Cancer Cell.

[14] Wang Y, Klijn JG, Zhang Y, Sieuwerts AM, Look MP (2005). Gene-expression profiles to predict distant metastasis of lymph-node-negative primary breast cancer.. Lancet.

[15] Pawitan Y, Bjohle J, Amler L, Borg AL, Egyhazi S (2005). Gene expression profiling spares early breast cancer patients from adjuvant therapy: derived and validated in two population-based cohorts.. Breast Cancer Res.

[16] Sorlie T, Perou CM, Tibshirani R, Aas T, Geisler S (2001). Gene expression pattern of breast carcinomas distinguish tumor subclasses with clinical implications.. PNAS.

[17] Choi JK, Yu U, Kim S, Yoo OJ (2003). Combining multiple microarray studies and modeling interstudy variation.. Bioinformatics.

[18] Lusa L, Gentleman R, Ruschhaupt M (2006). GeneMeta: MetaAnalysis for High Throughput Experiments.. R package version.

[19] Benjamini Y, Yekutieli D (2001). The control of the false discovery rate in multiple testing under dependency.. Ann Statist.

[20] Dahlquist KD, Salomonis N, Vranizan K, Lawlor SC, Conklin BR (2002). GenMAPP, a new tool for viewing and analyzing microarray data on biological pathways.. Nature Genetics.

[21] R website: www.R-package.org

[22] Bild AH, Yao G, Chang JT, Wang Q, Potti A (2006). Oncogenic pathway signatures in human cancers as a guide to targeted therapies.. Nature.

[23] Carroll JS, Meyer CA, Song J, Li W, Geistlinger TR (2006). Genome-wide analysis of estrogen receptor binding sites.. Nature Genetics.

[24] Musgrove EA, Sergio CM, Loi S, Inman CK, Anderson LR (2008). Identification of Functional Networks of Estrogen- and c-Myc-Responsive Genes and Their Relationship to Response to Tamoxifen Therapy in Breast Cancer.. PLoS ONE.

[25] Smyth GK, Gentleman R, Carey V, Dudoit S, Irizarry R, Huber W (2005). Limma: linear models for microarray data.. ‘Bioinformatics and Computational Biology Solutions using R and Bioconductor’.

[26] Xie X, Lu J, Kulbokas EJ, Golub TR, Mootha V (2005). Systematic discovery of regulatory motifs in human promoters and 3[prime] UTRs by comparison of several mammals.. Nature.

[27] Prall OW, Rogan EM, Musgrove EA, Watts CK, Sutherland RL (1998). c-Myc or cyclin D1 mimics estrogen effects on cyclin E-Cdk2 activation and cell cycle reentry.. Molecular and Cellular Biology.

[28] van't Veer LJ, Dai H, van de Vijver MJ, He YD, Hart AA (2002). Gene expression profiling predicts clinical outcome of breast cancer.. Nature.

[29] Jones C, Ford E, Gillett C, Ryder K, Merrett S (2004). Molecular Cytogenetic Identification of Subgroups of Grade III Invasive Ductal Breast Carcinomas with Different Clinical Outcomes.. Clin Cancer Res.

[30] Wang ZC, Lin M, Wei LJ, Li C, Miron A (2004). Loss of heterozygosity and its correlation with expression profiles in subclasses of invasive breast cancers.. Cancer Res.

[31] Bergamaschi A, Kim YH, Wang P, Sørlie T, Hernandez-Boussard T (2006). Distinct patterns of DNA copy number alteration are associated with different clinicopathological features and gene-expression subtypes of breast cancer.. Genes, Chromosomes and Cancer.

[32] Creighton CJ, Cordero KE, Larios JM, Miller RS, Johnson MD (2006). Genes regulated by estrogen in breast tumor cells in vitro are similarly regulated in vivo in tumor xenografts and human breast tumors.. Genome Biol.

[33] Zeller KI, Zhao X, Lee CWH, Chiu KP, Yao F (2006). Global mapping of c-Myc binding sites and target gene networks in human B cells.. PNAS.

[34] Rowland BD, Bernards R (2006). Re-Evaluating Cell-Cycle Regulation by E2Fs.. Cell.

[35] Vernell R, Helin K, Muller H (2003). Identification of Target Genes of the p16INK4A-pRB-E2F Pathway.. J Biol Chem.

[36] Black EP, Hallstrom T, Dressman HK, West M, Nevins JR (2005). Distinctions in the specificity of E2F function revealed by gene expression signatures.. Proceedings of the National Academy of Sciences.

[37] Balciunaite E, Spektor A, Lents NH, Cam H, te Riele H (2005). Pocket Protein Complexes Are Recruited to Distinct Targets in Quiescent and Proliferating Cells.. Mol Cell Biol.

[38] Ren B, Cam H, Takahashi Y, Volkert T, Terragni J (2002). E2F integrates cell cycle progression with DNA repair, replication, and G2/M checkpoints.. Genes Dev.

[39] Sorlie T, Wang Y, Xiao C, Johnsen H, Naume B (2006). Distinct molecular mechanisms underlying clinically relevant subtypes of breast cancer: Gene expression analyses across three different platforms.. BMC Genomics.

[40] Fulford LG, Easton DF, Reis-Filho JS, Sofronis A, Gillett CE (2006). Specific morphological features predictive for the basal phenotype in grade 3 invasive ductal carcinoma of breast.. Histopathology.

[41] Lin CY, Strom A, Vinsensius BV, Kong SL, Yeo AL (2004). Discovery of estrogen receptor α target genes and response elements in breast tumor cells.. Genome Biology.

[42] Cunliffe HE, Ringner M, Bilke S, Walker RL, Cheung JM (2003). The gene expression response of breast cancer to growth regulators: patterns and correlation with tumor expression profiles.. Cancer Res.

[43] McNeil CM, Sergio CM, Anderson LR, Inman CK, Eggleton SA (2006). c-Myc overexpression and endocrine resistance in breast cancer.. The Journal of Steroid Biochemistry and Molecular Biology.

[44] Naidu R, Wahab NA, Yadav M, Kutty MK (2002). Protein expression and molecular analysis of c-myc gene in primary breast carcinomas using immunohistochemistry and differential polymerase chain reaction.. Int J Mol Med.

[45] Cuny M, Kramar A, Courjal F, Johannsdottir V, Iacopetta B (2000). Relating Genotype and Phenotype in Breast Cancer: An Analysis of the Prognostic Significance of Amplification at Eight Different Genes or Loci and of p53 Mutations.. Cancer Res.

[46] Rodriguez-Pinilla SM, Jones RL, Lambros MB, Arriola E, Savage K (2006). MYC amplification in breast cancer: a chromogenic in situ hybridisation study.. J Clin Pathol.

[47] Blancato J, Singh B, Liu A, Liao DJ, Dickson RB (2004). Correlation of amplification and overexpression of the c-myc oncogene in high-grade breast cancer: FISH, in situ hybridisation and immunohistochemical analyses.. Br J Cancer.

[48] Rhodes DR, Kalyana-Sundaram S, Tomlins SA, Mahavisno V, Kasper N (2007). Molecular concepts analysis links tumors, pathways, mechanisms, and drugs.. Neoplasia.

[49] Adem C, Soderberg CL, Hafner K, Reynolds CA, Slezak JM (2004). *ERBB2*,*TBX2*, *RPS6KB1*, and *MYC* alterations in breast tissues of *BRCA1* and *BRCA2* mutation carriers.. Genes, Chromosomes and Cancer.

[50] Efstratiadis A, Szabolcs M, Klinakis A (2007). Notch, Myc and Breast Cancer.. Cell Cycle.

[51] Leone G, Sears R, Huang E, Rempel R, Nuckolls F (2001). Myc Requires Distinct E2F Activities to Induce S Phase and Apoptosis.. Molecular Cell.

[52] Ogawa H, Ishiguro K-I, Gaubatz S, Livingston DM, Nakatani Y (2002). A Complex with Chromatin Modifiers That Occupies E2F- and Myc-Responsive Genes in G0 Cells.. Science.

[53] Kreike B, van Kouwenhove M, Horlings H, Weigelt B, Peterse H (2007). Gene expression profiling and histopathological characterization of triple-negative/basal-like breast carcinomas.. Breast Cancer Res.

[54] Yu D, Cozma D, Park A, Thomas-Tikhonenko A (2005). Functional validation of genes implicated in lymphomagenesis: an in vivo selection assay using a Myc-induced B-cell tumor.. Ann N Y Acad Sci.

