# Supplementary figures and images for: Meta-Analysis and Gene Set Enrichment Relative to ER Status Reveal Elevated Activity of MYC and E2F in the “Basal” Breast Cancer Subgroup
Source: PLoS One. 2009 Mar 9;4(3):e4710. doi: 10.1371/journal.pone.0004710 (PMC2650420; doi:10.1371/journal.pone.0004710)

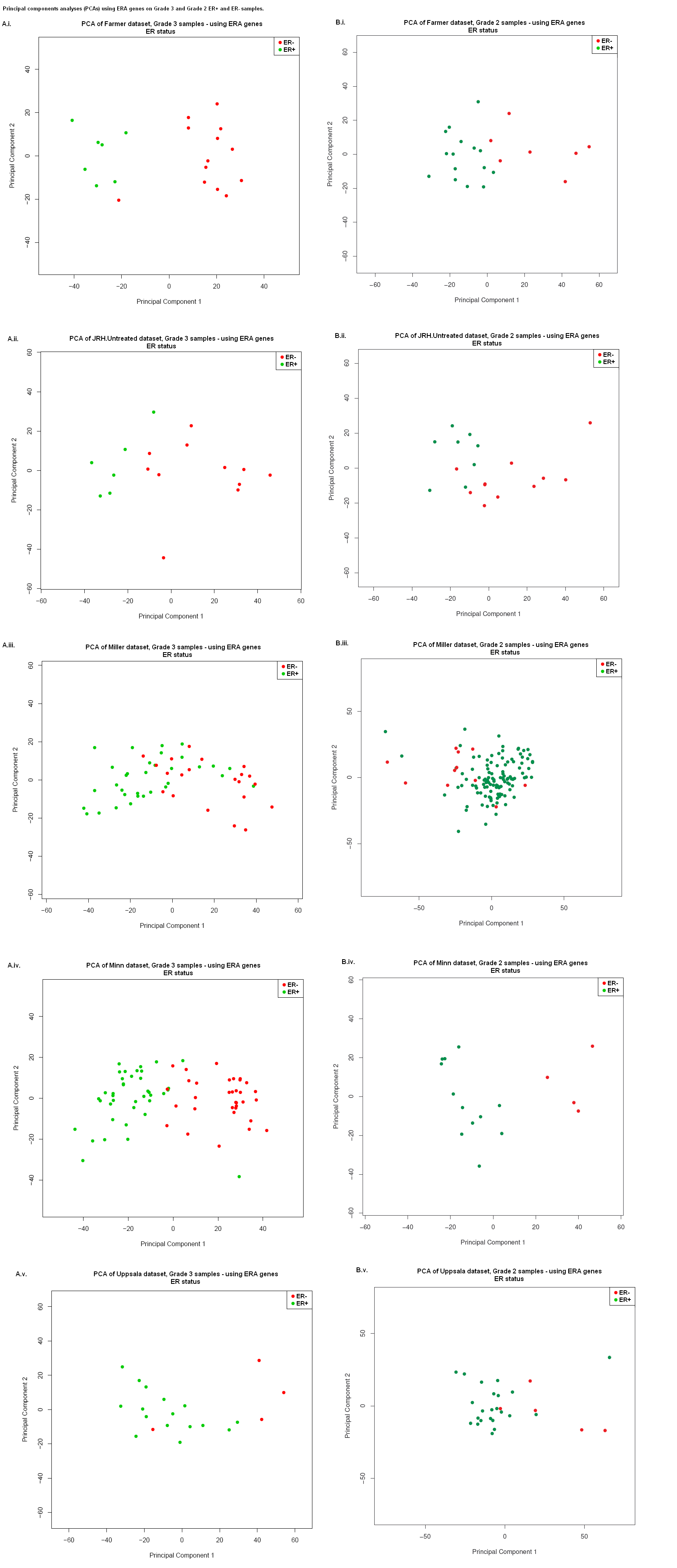

Supplement: Figure S1 — Principal components analyses (PCAs) using ERA genes on Grade 3 and Grade 2 ER+ and ER− samples. The ERA genes were identified by a meta-analysis of Grade 3 samples in five datasets (Farmer, Sotiriou.JRH.Untreated, Miller, Minn and Sotiriou.Uppsala). To demonstrate that these ERA genes also separate Grade 2 ER+ and ER− tumors, PCAs using the ERA genes were performed on Grade 2 samples from those same five datasets. The PCAs of Grade 3 samples in the Farmer, Sotiriou.JRH.Untreated, Miller, Minn and Sotiriou.Uppsala datasets are found in Figure S1A.i–v; PCAs of Grade 2 samples in the Farmer, Sotiriou.JRH.Untreated, Miller, Minn and Sotiriou.Uppsala datasets are found in Figure S1B.i–v. (0.54 MB TIF) [file pone.0004710.s002.tif]

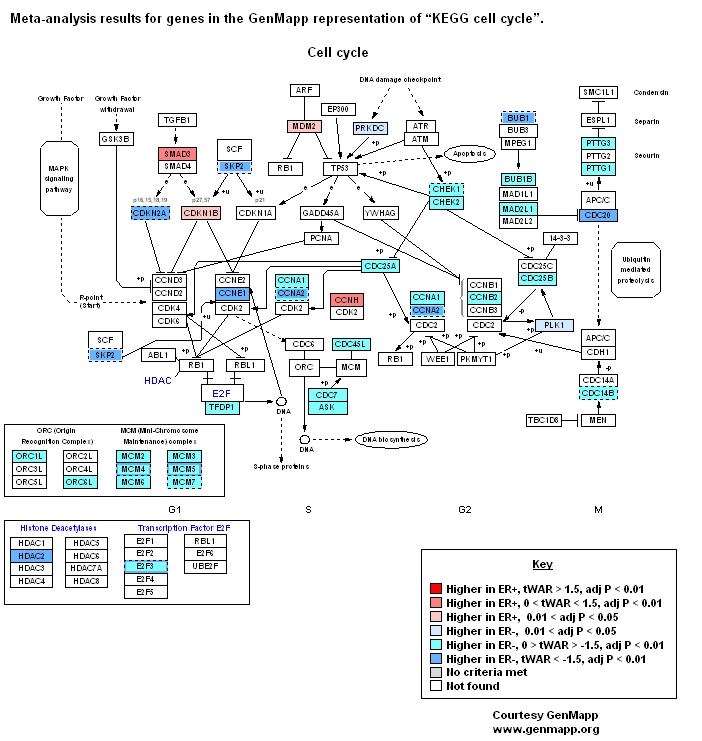

Supplement: Figure S2 — Meta-analysis results for genes in the GenMapp representation of KEGG Cell Cycle. Genes are colored by whether they have higher expression in ER+ or ER tumors from the meta-analysis, with the foldchange and significance of this over-expression represented by the transformed Weighted Average Ratio (tWAR) and BY-adjusted P-value (adj P) respectively. (0.08 MB TIF) [file pone.0004710.s003.tif]

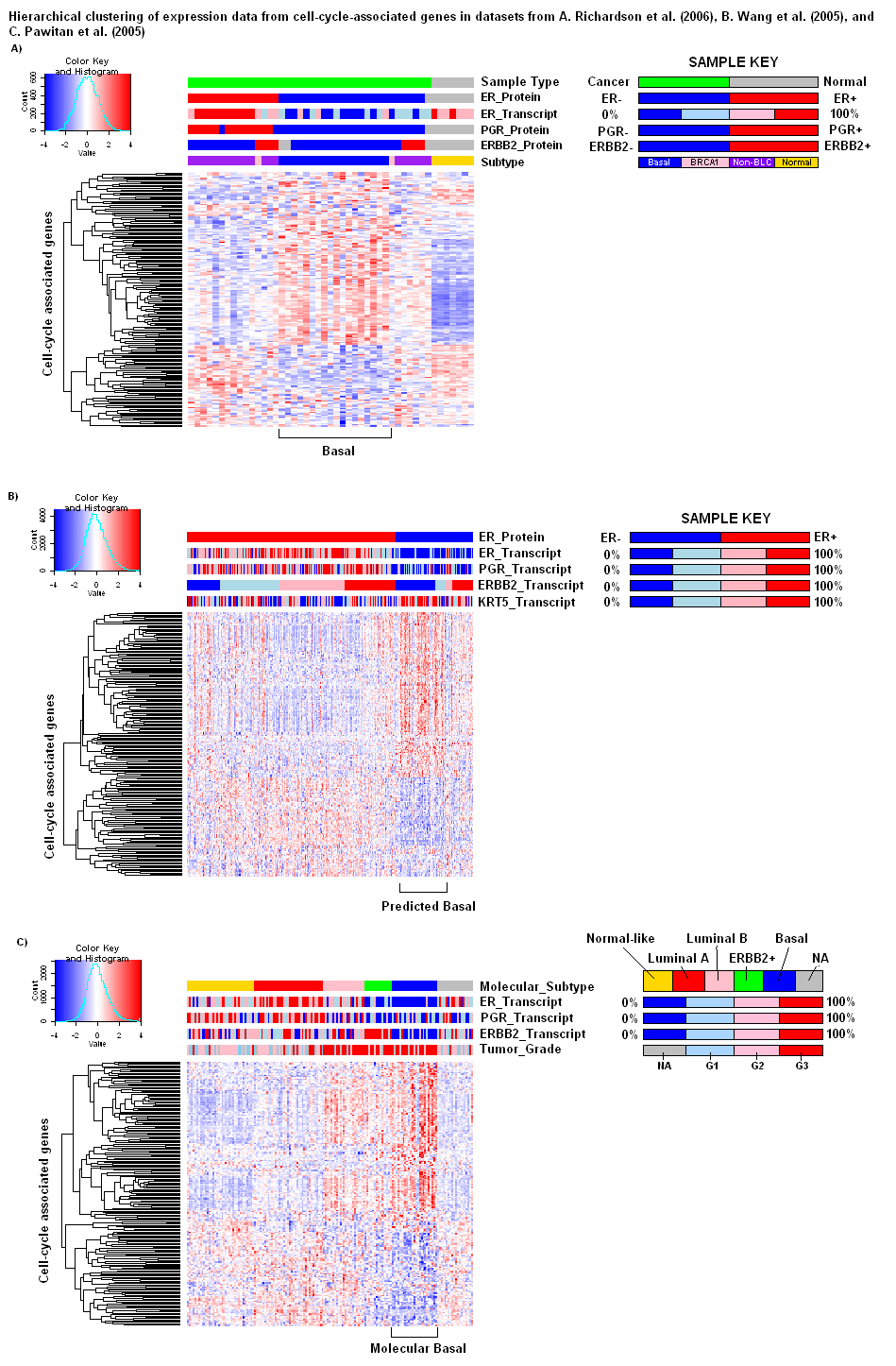

Supplement: Figure S3 — Hierarchical clustering of expression data from cell-cycle-associated genes in datasets from A. Richardson et al. (2006), B. Wang et al. (2005), and C. Pawitan et al. (2005). The two-way clustering of the ERA genes and samples previously (Figure 1) had indicated that the ERA genes were differentially expressed between the ER+, basal and ER−/ERBB2+ tumor subtypes. In order to clarify the behavior of the cell-cycle-associated genes in the different subtypes, we ordered the samples in the validation datasets primarily by ER status and secondarily by ERBB2 status, and then clustered only the cell-cycle-associated genes while maintaining the order of the samples. Figure S3A data is derived from the Richardson dataset (Richardson et al., 2006) and the top color bars indicate the following: “Sample Type” - whether the sample is a breast cancer or from normal breast tissue; “ER_Protein” - ER status determined using IHC; “PGR_Protein” - PGR status determined using IHC; “ERBB2_Protein” - ERBB2 status determined using IHC; “Subtype” - Subtype determined from IHC results (Basal, BRCA1 mutation positive, non-basal-like carcinoma (“Non-BLC”), or Normal tissue) (see sample key). Figure S3B. data is derived from the Wang dataset (Wang et al. 2005) and the top color bars represent the following: “ER_Protein” - ER status as determined using ligand binding assay or IHC; “ER_Transcript”, “PGR_Transcript”, “ERBB2_Transcript” and “KRT5_Transcript”: relative expression measured from quantiles of probe set intensities as described in “Data Collection” in Materials and Methods (see sample key). Figure S3C. data is derived from the Pawitan dataset (Pawitan et al. 2005) and the top color bars represent the following: “Molecular_Subtype” - determined by correlation to the normal-like, Luminal A, Luminal B, ERBB2+ and basal molecular subtypes (Sorlie et al. 2001); “ER_Transcript”, “PGR_Transcript”, and “ERBB2_Transcript” as above; and “Tumor_Grade” - Elston Ellis grading (see sample key). Two m [file pone.0004710.s004.tif]

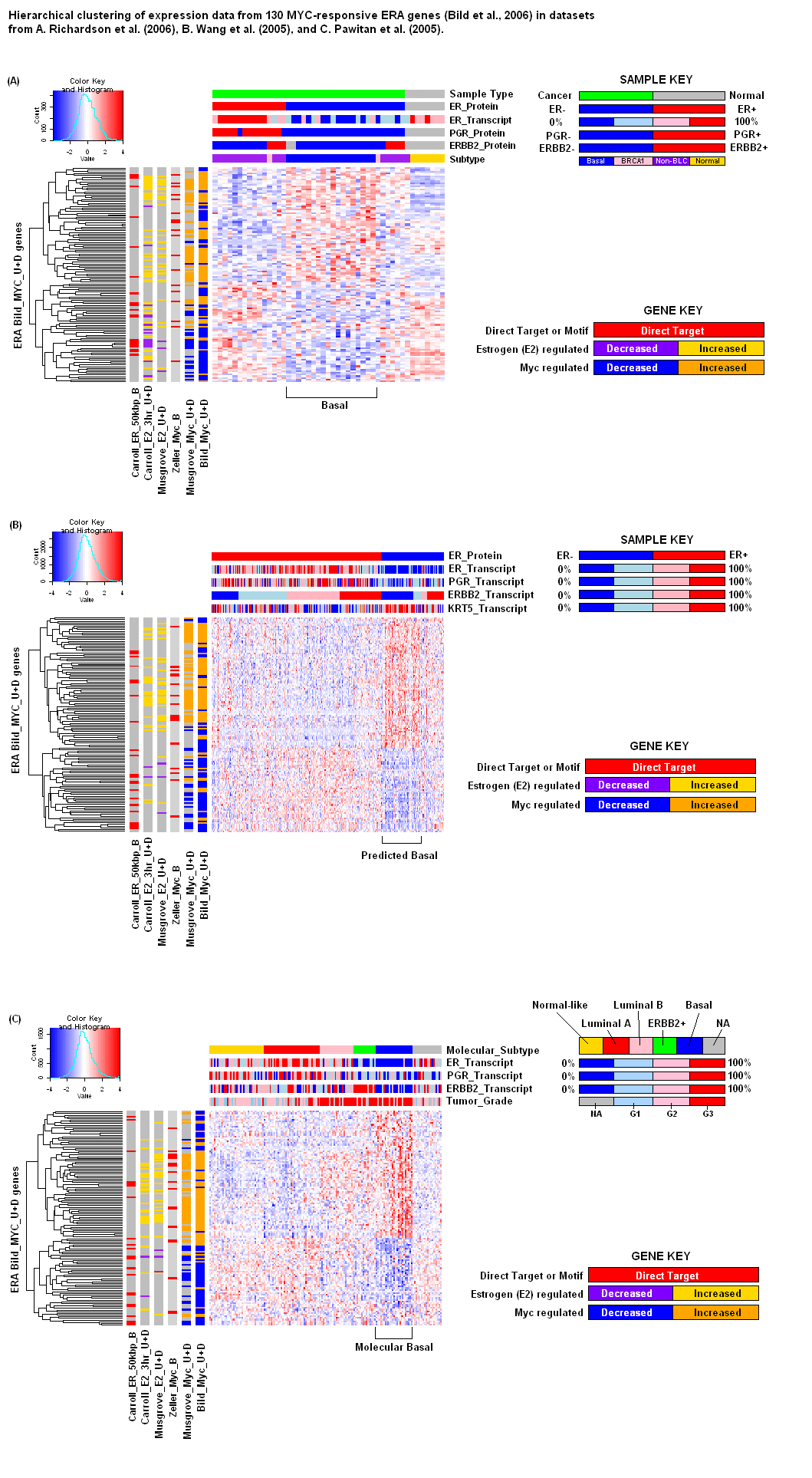

Supplement: Figure S4 — Hierarchical clustering of expression data from 130 MYC-responsive ERA genes (Bild et al., 2006) in datasets from A. Richardson et al. (2006), B. Wang et al. (2005), and C. Pawitan et al. (2005). The ERA Bild_MYC_U+D genes are ERA genes that were also regulated by MYC in HMECs (Bild et al. 2006). We clustered these genes in the three validation datasets while maintaining the sample order. In Figure S4A, the top color bars are equivalent to those of Figure S3A. The colors in the side color bars represent the following: yellow = an E2-induced gene, purple = an E2-repressed gene, orange = a MYC-induced gene, blue = a MYC-repressed gene and red indicates a gene which is a direct target of ER (“ER_B” suffix), or a direct target of MYC (“Myc_B”). Moving from right to left, for each gene, the first two side color bars represent the transcriptional response to MYC in HMECs (Bild et al., 2006) (Bild_MYC_U+D), and in MCF-7 cells (Musgrove et al., 2008; McNeil et al., 2006) (Musgrove_Myc_U+D). The next color bar represents whether the gene was classified as being a direct target of MYC in B cell lymphomas (Zeller et al., 2006) (Zeller_Myc_B). The next three color bars represent how this gene was regulated by E2 in MCF-7 cells at 6 hours (Musgrove et al., 2008; McNeil et al., 2006) (Musgrove_E2_U+D), and at 3 hours (Carroll et al., 2006) (Carroll_3 hr_E2_U+D), and whether it contained an ER-binding site with 50 kbp of the promoter region (Carroll et al., 2006) (Carroll_ER_50 kbp_B). The top color bars of Figure S4B are equivalent to those of Figure S3B, and the side color bars are the counterparts of those in Figure S4A. The top color bars of Figure S4C are equivalent to those of Figure S3C, and the side color bars are the counterparts of those in Figure S4A. It can be seen that the majority of ERA MYC-induced genes have higher expression in the basal tumors, and the majority of ERA MYC-repressed genes have lower expression in basal tumors. (0.48 MB TIF) [file pone.0004710.s005.tif]

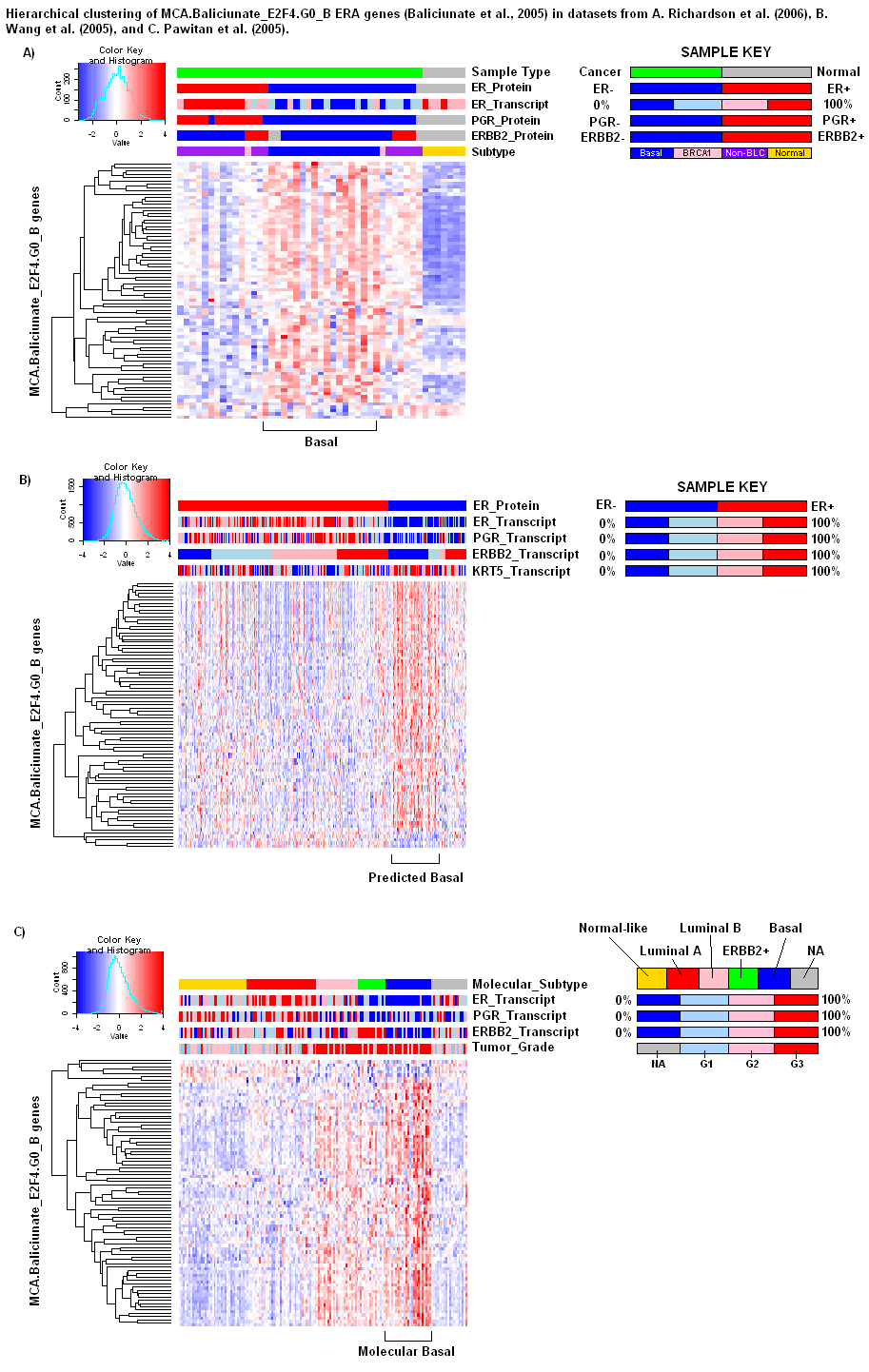

Supplement: Figure S5 — Hierarchical clustering of MCA.Baliciunate_E2F4.G0_B ERA genes (Baliciunate et al., 2005) in datasets from A. Richardson et al. (2006), B. Wang et al. (2005), and C. Pawitan et al. (2005). The MCA.Baliciunate_E2F4.G0_B genes are direct targets of E2F4 in mouse embryonic fibroblasts (Baliciunate et al., 2005). In Figure S5, the top color bars are equivalent of Figure S5A, S5B and S5C are equivalent to those of to those of Figure S3A, S3B and S3C respectively. (0.35 MB TIF) [file pone.0004710.s006.tif]
