# Supplementary material for: Meta-Analysis and Gene Set Enrichment Relative to ER Status Reveal Elevated Activity of MYC and E2F in the “Basal” Breast Cancer Subgroup
Source: PLoS One. 2009 Mar 9;4(3):e4710. doi: 10.1371/journal.pone.0004710 (PMC2650420; doi:10.1371/journal.pone.0004710)
Supplement: Table S2 — Cell cycle genes over-expressed in ER− or ER+ tumors. Cell cycle associated genes, and their over-expression in ER− tumors or ER+ tumors from the meta-analysis. (0.02 MB PDF) [file pone.0004710.s008.pdf]

Additional.File.4.xls - Cell cycle genes over-expressed in ER- or ER+ tumors

Cell cycle associated genes, and their over-expression in ER- tumors or ER+ tumors from the meta-analysis.

Probe Set ID	Gene Title	Gene Symbol	Chromosomal Location	Over-expressed in ER+ or ER-	Adjusted P
218575_at	anaphase promoting complex subunit 1	ANAPC1	chr2q12.1	ER-	0.002
213419_at	amyloid beta (A4) precursor protein-binding, family B, member 2 (Fe	APBB2	chr4p14	ER+	0.001
202268_s_at	amyloid beta precursor protein binding protein 1	APPBP1	chr16q22	ER-	0.002
218158_s_at	adaptor protein containing pH domain, PTB domain and leucine zipp	APPL	chr3p21.1-p14.3	ER+	0.000
204244_s_at	activator of S phase kinase	ASK	chr7q21.3	ER-	0.000
219918_s_at	asp (abnormal spindle)-like, microcephaly associated (Drosophila)	ASPM	chr1q31	ER-	0.000
209464_at	aurora kinase B	AURKB	chr17p13.1	ER-	0.000
210201_x_at	bridging integrator 1	BIN1	chr2q14	ER-	0.009
202094_at	baculoviral IAP repeat-containing 5 (survivin)	BIRC5	chr17q25	ER-	0.000
212949_at	barren homolog (Drosophila)	BRRN1	chr2q11.2	ER-	0.000
205548_s_at	BTG family, member 3	BTG3	chr21q21.1-q21.2	ER-	0.000
209642_at	BUB1 budding uninhibited by benzimidazoles 1 homolog (yeast)	BUB1	chr2q14	ER-	0.000
203755_at	BUB1 budding uninhibited by benzimidazoles 1 homolog beta (yeast	BUB1B	chr15q15	ER-	0.000
201725_at	chromosome 10 open reading frame 7	C10orf7	chr10p13	ER-	0.000
205899_at	cyclin A1	CCNA1	chr13q12.3-q13	ER-	0.000
213226_at	Cyclin A2	CCNA2	chr4q25-q31	ER-	0.000
202705_at	cyclin B2	CCNB2	chr15q22.2	ER-	0.000
208712_at	cyclin D1	CCND1	chr11q13	ER+	0.000
213523_at	cyclin E1	CCNE1	chr19q12	ER-	0.000
202770_s_at	cyclin G2	CCNG2	chr4q21.1	ER+	0.000
204093_at	cyclin H	CCNH	chr5q13.3-q14	ER+	0.001
219470_x_at	cyclin J	CCNJ	chr10pter-q26.12	ER-	0.000
221555_x_at	CDC14 cell division cycle 14 homolog B (S. cerevisiae)	CDC14B	chr9q22.33	ER-	0.000
202870_s_at	CDC20 cell division cycle 20 homolog (S. cerevisiae)	CDC20	chr1p34.1	ER-	0.000
204695_at	cell division cycle 25A	CDC25A	chr3p21	ER-	0.000
201853_s_at	cell division cycle 25B	CDC25B	chr20p13	ER-	0.000
217878_s_at	cell division cycle 27	CDC27	chr17q12-17q23.2	ER-	0.002
204126_s_at	CDC45 cell division cycle 45-like (S. cerevisiae)	CDC45L	chr22q11.21	ER-	0.000
204510_at	CDC7 cell division cycle 7 (S. cerevisiae)	CDC7	chr1p22	ER-	0.008
221520_s_at	cell division cycle associated 8	CDCA8	chr1p34.3	ER-	0.000
201938_at	CDK2-associated protein 1	CDK2AP1	chr12q24.31	ER-	0.000
211297_s_at	cyclin-dependent kinase 7 (MO15 homolog, Xenopus laevis, cdk-act	CDK7	chr5q12.1	ER+	0.010
209644_x_at	cyclin-dependent kinase inhibitor 2A (melanoma, p16, inhibits CDK4	CDKN2A	chr9p21	ER-	0.000
205046_at	centromere protein E, 312kDa	CENPE	chr4q24-q25	ER-	0.009
207331_at	centromere protein F, 350/400ka (mitosin)	CENPF	chr1q32-q41	ER-	0.000
204605_at	cell growth regulator with ring finger domain 1	CGRRF1	chr14q22.2	ER+	0.000

204775_at	chromatin assembly factor 1, subunit B (p60)	CHAF1B	chr21q22.13	ER-	0.001
205394_at	CHK1 checkpoint homolog (S. pombe)	CHEK1	chr11q24-q24	ER-	0.000
210416_s_at	CHK2 checkpoint homolog (S. pombe)	CHEK2	chr22q11|22q12.1	ER-	0.009
201897_s_at	CDC28 protein kinase regulatory subunit 1B	CKS1B	chr1q21.2	ER-	0.000
204170_s_at	CDC28 protein kinase regulatory subunit 2	CKS2	chr9q22	ER-	0.000
212308_at	cytoplasmic linker associated protein 2	CLASP2	chr3p23	ER+	0.007
201774_s_at	chromosome condensation-related SMC-associated protein 1	CNAP1	chr12p13.3	ER-	0.006
207614_s_at	cullin 1	CUL1	chr7q36.1	ER-	0.009
209665_at	cytochrome b-561 domain containing 2	CYB561D2	chr3p21.3	ER+	0.000
208149_x_at	DEAD/H (Asp-Glu-Ala-Asp/His) box polypeptide 11 (CHL1-like helica	DDX11	chr12p11	ER-	0.003
218218_at	DIP13 beta	DIP13B	chr12q24.1	ER+	0.000
201681_s_at	discs, large homolog 5 (Drosophila)	DLG5	chr10q23	ER+	0.002
210469_at	discs, large homolog 5 (Drosophila)	DLG5	chr10q23	ER+	0.001
203764_at	discs, large homolog 7 (Drosophila)	DLG7	chr14q22.3	ER-	0.001
204014_at	dual specificity phosphatase 4	DUSP4	chr8p12-p11	ER+	0.001
203693_s_at	E2F transcription factor 3	E2F3	chr6p22	ER-	0.000
219454_at	EGF-like-domain, multiple 6	EGFL6	chrXp22	ER-	0.000
201983_s_at	epidermal growth factor receptor (erythroblastic leukemia viral (v-erb	EGFR	chr7p12	ER-	0.000
217941_s_at	erbb2 interacting protein	ERBB2IP	chr5q12.3	ER+	0.002
205225_at	estrogen receptor 1	ESR1	chr6q25.1	ER+	0.000
201995_at	exostoses (multiple) 1	EXT1	chr8q24.11-q24.1:	ER-	0.005
203989_x_at	coagulation factor II (thrombin) receptor	F2R	chr5q13	ER+	0.000
206404_at	fibroblast growth factor 9 (glia-activating factor)	FGF9	chr13q11-q12	ER-	0.009
212985_at	Hypothetical protein FLJ14001	FLJ14001	chr4p14	ER+	0.009
202281_at	cyclin G associated kinase	GAK	chr4p16	ER+	0.007
218350_s_at	geminin, DNA replication inhibitor	GMNN	chr6p22.2	ER-	0.000
202045_s_at	glucocorticoid receptor DNA binding factor 1	GRLF1	chr19q13.3	ER+	0.002
204318_s_at	G-2 and S-phase expressed 1	GTSE1	chr22q13.2-q13.3	ER-	0.000
204317_at	G-2 and S-phase expressed 1 /// hypothetical gene supported by BC	GTSE1 /// LOC4	chr22q13.2-q13.3	ER-	0.000
218662_s_at	chromosome condensation protein G	HCAP-G	chr4p15.33	ER-	0.000
201833_at	histone deacetylase 2	HDAC2	chr6q21	ER-	0.000
205659_at	histone deacetylase 9	HDAC9	chr7p21.1	ER-	0.006
202815_s_at	hexamethylene bis-acetamide inducible 1	HEXIM1	chr17q21.31	ER+	0.000
210719_s_at	high-mobility group 20B	HMG20B	chr19p13.3	ER+	0.001
209581_at	HRAS-like suppressor 3	HRASLS3	chr11q12.3-q13.1	ER+	0.000
211538_s_at	heat shock 70kDa protein 2	HSPA2	chr14q24.1	ER+	0.001
206341_at	interleukin 2 receptor, alpha	IL2RA	chr10p15-p14	ER-	0.007
202859_x_at	interleukin 8	IL8	chr4q13-q21	ER-	0.005
208930_s_at	interleukin enhancer binding factor 3, 90kDa	ILF3	chr19p13.2	ER-	0.009
204444_at	kinesin family member 11	KIF11	chr10q24.1	ER-	0.006
209408_at	kinesin family member 2C	KIF2C	chr1p34.1	ER-	0.000
209680_s_at	kinesin family member C1	KIFC1	chr6p21.3	ER-	0.000
204162_at	kinetochore associated 2	KNTC2	chr18p11.32	ER-	0.000

221833_at	Peroxisomal LON protease like	LONPL	chr16q12.1	ER+	0.002
203362_s_at	MAD2 mitotic arrest deficient-like 1 (yeast)	MAD2L1	chr4q27	ER-	0.001
205698_s_at	mitogen-activated protein kinase kinase 6	MAP2K6	chr17q24.3	ER-	0.009
202501_at	microtubule-associated protein, RP/EB family, member 2	MAPRE2	chr18q12.1	ER-	0.001
202107_s_at	MCM2 minichromosome maintenance deficient 2, mitotin (S. cerevis	MCM2	chr3q21	ER-	0.003
201555_at	MCM3 minichromosome maintenance deficient 3 (S. cerevisiae)	MCM3	chr6p12	ER-	0.004
222037_at	MCM4 minichromosome maintenance deficient 4 (S. cerevisiae)	MCM4	chr8q11.2	ER-	0.000
216237_s_at	MCM5 minichromosome maintenance deficient 5, cell division cycle	MCM5	chr22q13.1	ER-	0.000
201930_at	MCM6 minichromosome maintenance deficient 6 (MIS5 homolog, S.	MCM6	chr2q21	ER-	0.000
208795_s_at	MCM7 minichromosome maintenance deficient 7 (S. cerevisiae)	MCM7	chr7q21.3-q22.1	ER-	0.000
202556_s_at	microspherule protein 1	MCRS1	chr12q13.12	ER+	0.006
212022_s_at	antigen identified by monoclonal antibody Ki-67	MKI67	chr10q25-qter	ER-	0.000
202520_s_at	mutL homolog 1, colon cancer, nonpolyposis type 2 (E. coli)	MLH1	chr3p21.3	ER+	0.002
203565_s_at	menage a trois 1 (CAK assembly factor)	MNAT1	chr14q23	ER+	0.003
205235_s_at	M-phase phosphoprotein 1	MPHOSPH1	chr10q23.31	ER-	0.008
209421_at	mutS homolog 2, colon cancer, nonpolyposis type 1 (E. coli)	MSH2	chr2p22-p21	ER-	0.010
204798_at	v-myb myeloblastosis viral oncogene homolog (avian)	MYB	chr6q22-q23	ER+	0.002
201710_at	v-myb myeloblastosis viral oncogene homolog (avian)-like 2	MYBL2	chr20q13.1	ER-	0.000
201621_at	neuroblastoma, suppression of tumorigenicity 1	NBL1	chr1p36.13-p36.1	ER+	0.005
219542_at	NIMA (never in mitosis gene a)- related kinase 11	NEK11	chr3q21.3	ER+	0.000
212678_at	Neurofibromin 1 (neurofibromatosis, von Recklinghausen disease, W	NF1	chr17q11.2	ER+	0.010
210767_at	neurofibromin 2 (bilateral acoustic neuroma)	NF2	chr22q12.2	ER-	0.002
201077_s_at	NHP2 non-histone chromosome protein 2-like 1 (S. cerevisiae)	NHP2L1	chr22q13.2-q13.3	ER-	0.001
214427_at	nucleolar protein 1, 120kDa	NOL1	chr12p13	ER-	0.000
205085_at	origin recognition complex, subunit 1-like (yeast)	ORC1L	chr1p32	ER-	0.000
219105_x_at	origin recognition complex, subunit 6 homolog-like (yeast)	ORC6L	chr16q12	ER-	0.000
210094_s_at	par-3 partitioning defective 3 homolog (C. elegans)	PARD3	chr10p11.22-p11.	ER-	0.001
221526_x_at	par-3 partitioning defective 3 homolog (C. elegans)	PARD3	chr10p11.22-p11.	ER-	0.002
208823_s_at	PCTAIRE protein kinase 1	PCTK1	chrXp11.3-p11.23	ER-	0.006
221918_at	PCTAIRE protein kinase 2	PCTK2	chr12q23.1	ER+	0.001
204886_at	polo-like kinase 4 (Drosophila)	PLK4	chr4q27-q28	ER-	0.002
209640_at	promyelocytic leukemia	PML	chr15q22	ER-	0.000
221206_at	PMS2 postmeiotic segregation increased 2 (S. cerevisiae)	PMS2	chr7p22.2	ER+	0.001
202466_at	polymerase (DNA directed) sigma	POLS	chr5p15	ER-	0.000
201407_s_at	protein phosphatase 1, catalytic subunit, beta isoform	PPP1CB	chr2p23	ER-	0.002
208652_at	protein phosphatase 2 (formerly 2A), catalytic subunit, alpha isoform	PPP2CA	chr5q31.1	ER+	0.000
202432_at	protein phosphatase 3 (formerly 2B), catalytic subunit, beta isoform	PPP3CB	chr10q21-q22	ER+	0.009
218009_s_at	protein regulator of cytokinesis 1	PRC1	chr15q26.1	ER-	0.002
213093_at	protein kinase C, alpha	PRKCA	chr17q22-q23.2	ER-	0.000
209815_at	patched homolog (Drosophila)	PTCH	chr9q22.3	ER-	0.003
203554_x_at	pituitary tumor-transforming 1	PTTG1	chr5q35.1	ER-	0.000
210826_x_at	RAD17 homolog (S. pombe)	RAD17	chr5q13	ER+	0.000
209349_at	RAD50 homolog (S. cerevisiae)	RAD50	chr5q31	ER+	0.001

205024_s_at	RAD51 homolog (RecA homolog, E. coli) (S. cerevisiae)	RAD51	chr15q15.1	ER-	0.001
204558_at	RAD54-like (S. cerevisiae)	RAD54L	chr1p32	ER-	0.000
202677_at	RAS p21 protein activator (GTPase activating protein) 1	RASA1	chr5q13.3	ER+	0.000
212331_at	retinoblastoma-like 2 (p130)	RBL2	chr16q12.2	ER+	0.005
201394_s_at	RNA binding motif protein 5	RBM5	chr3p21.3	ER+	0.001
206499_s_at	regulator of chromosome condensation 1	RCC1	chr1p36.1	ER-	0.000
212099_at	ras homolog gene family, member B	RHOB	chr2p24	ER+	0.000
201003_x_at	arginyl aminopeptidase (aminopeptidase B) /// ubiquitin-conjugating	RNPEP /// UBE2	chr1q32 /// chr20q	ER+	0.007
220425_x_at	ropporin, rhophilin associated protein 1B	ROPN1B	chr3q21.2	ER-	0.000
208999_at	septin 8	SEP8	chr5q31	ER+	0.003
209339_at	seven in absentia homolog 2 (Drosophila) /// seven in absentia homo	SIAH2	chr3q25	ER+	0.000
200719_at	S-phase kinase-associated protein 1A (p19A)	SKP1A	chr5q31	ER+	0.000
203625_x_at	S-phase kinase-associated protein 2 (p45)	SKP2	chr5p13	ER-	0.000
205398_s_at	SMAD, mothers against DPP homolog 3 (Drosophila)	SMAD3	chr15q22.33	ER+	0.003
204240_s_at	SMC2 structural maintenance of chromosomes 2-like 1 (yeast)	SMC2L1	chr9q31.1	ER-	0.004
201664_at	SMC4 structural maintenance of chromosomes 4-like 1 (yeast)	SMC4L1	chr3q26.1	ER-	0.005
202764_at	stromal interaction molecule 1	STIM1	chr11p15.5	ER-	0.001
208079_s_at	serine/threonine kinase 6	STK6	chr20q13.2-q13.3	ER-	0.001
200783_s_at	stathmin 1/oncoprotein 18	STMN1	chr1p36.1-p35	ER-	0.000
204496_at	striatin, calmodulin binding protein 3	STRN3	chr14q13-q21	ER+	0.001
212330_at	transcription factor Dp-1	TFDP1	chr13q34	ER-	0.007
203588_s_at	transcription factor Dp-2 (E2F dimerization partner 2)	TFDP2	chr3q23	ER-	0.002
205016_at	transforming growth factor, alpha	TGFA	chr2p13	ER-	0.000
203120_at	tumor protein p53 binding protein, 2	TP53BP2	chr1q42.1	ER-	0.000
210052_s_at	TPX2, microtubule-associated, homolog (Xenopus laevis)	TPX2	chr20q11.2	ER-	0.000
204822_at	TTK protein kinase	TTK	chr6q13-q21	ER-	0.000
203273_s_at	tumor suppressor candidate 2	TUSC2	chr3p21.3	ER+	0.001
203246_s_at	tumor suppressor candidate 4	TUSC4	chr3p21.3	ER+	0.002
202835_at	thioredoxin-like 4A	TXNL4A	chr18q23	ER-	0.000
203614_at	UTP14, U3 small nucleolar ribonucleoprotein, homolog C (yeast)	UTP14C	chr13q14.2	ER+	0.005
203683_s_at	vascular endothelial growth factor B	VEGFB	chr11q13	ER+	0.004
217717_s_at	tyrosine 3-monooxygenase/tryptophan 5-monooxygenase activation	YWHAB	chr20q13.1	ER+	0.001
213699_s_at	tyrosine 3-monooxygenase/tryptophan 5-monooxygenase activation	YWHAQ /// MIB1	chr2p25.1 /// chr18	ER-	0.008
