# Supplementary material for: Meta-Analysis and Gene Set Enrichment Relative to ER Status Reveal Elevated Activity of MYC and E2F in the “Basal” Breast Cancer Subgroup
Source: PLoS One. 2009 Mar 9;4(3):e4710. doi: 10.1371/journal.pone.0004710 (PMC2650420; doi:10.1371/journal.pone.0004710)
Supplement: Text S1 — Supplementary Methods (0.10 MB DOC) [file pone.0004710.s009.doc]

# Supporting Information Text S1.

# Supplementary Materials and Methods

[Additional file 1. Supplementary Materials and Methods 1](#__RefHeading___Toc191181583)

[Patient characteristics 2](#__RefHeading___Toc191181584)

[Meta-analysis methods 3](#__RefHeading___Toc191181585)

[Weighted average ratio 3](#__RefHeading___Toc191181586)

[Functional analysis 4](#__RefHeading___Toc191181587)

[References: 5](#__RefHeading___Toc191181588)

## Patient characteristics

A. Grade 3 datasets used in the meta-analysis

| **Dataset** | **Total # of samples** | **ER status by IHC (ER+/ ER-)** | **ERBB2 status by IHC**  **(Pos /**  **Neg /**  **NA)** | **Lymph Node Status (Pos /**  **Neg /**  **NA)** | **Treatment Status**  **(Chemotherapy/**  **Endocrine / None /**  **NA)** | **Dichotomized size**  **(≤20mm /**  **>20mm /**  **NA)** |
| --- | --- | --- | --- | --- | --- | --- |
| Farmer.G3  [1] | 20 | ER+  n = 7 | 0/0/7 | 0/0/7 | 0/0/0/7 | 0/0/7 |
|  |  | ER-  n = 13 | 0/0/13 | 0/0/13 | 0/0/0/13 | 0/0/13 |
| JRH.Untreated.G3 [2] | 17 | ER+  n= 6 | 0/0/6 | 0/6/0 | 0/0/6/0 | 2/4/0 |
|  |  | ER-  n = 11 | 0/0/11 | 11/0/0 | 0/0/11/0 | 3/8/0 |
| Miller.G3  [3] | 54 | ER+  n = 33 | 0/0/33 | 19/12/2 | 9/13/10/1 | 6/27/0 |
|  |  | ER-  n = 21 | 0/0/21 | 9/10/2 | 3/7/11/0 | 4/17/0 |
| Minn.G3  [4] | 72 | ER+  n = 39 | 10/26/3 | 14/25/0 | 0/0/0/39 | 5/34/0 |
|  |  | ER-  n = 33 | 11/20/2 | 15/18/0 | 0/0/0/33 | 5/28/0 |
| Uppsala.G3  [2] | 20 | ER+  n = 16 | 0/0/16 | 8/7/1 | 0/10/6/0 | 1/15/0 |
|  |  | ER-  n = 4 | 0/0/4 | 0/4/0 | 0/0/4/0 | 2/2/0 |

B. Independent datasets used for validation

| **Dataset** | **Total # of samples** | **ER status by IHC (ER+/ ER-)** | **ERBB2 status by IHC**  **(Pos /**  **Neg /**  **NA)** | **Lymph Node Status**  **(Pos /**  **Neg /**  **NA)** | **Tumor Grade (G1/ G2/ G3/ NA)** | **Treatment Status**  **(Available/ NA)** | **Size**  **(Available/ NA)** |
| --- | --- | --- | --- | --- | --- | --- | --- |
| Richardson  [5] | 47 | ER+  n = 15 | 4/11/0 | 0/0/15 | 0/0/15/0 | 0/47 | 0/47 |
|  |  | ER-  n = 24 | 4/18/2 | 0/0/24 | 0/0/24/0 | 0/24 | 0/24 |
|  |  | NA  n = 1 | 0/0/1 | 0/0/1 | 0/0/1/0 | 0/1 | 0/1 |
|  |  | Normal breast  n = 7 | 0/0/7 | 0/0/7 | 0/0/0/7 | 0/7 | 0/7 |
| Wang  [6] | 286 | ER+  n = 209 | 0/0/209 | 0/209/0 | 0/0/0/209 | 0/209 | 0/209 |
|  |  | ER-  n = 77 | 0/0/77 | 0/77/0 | 0/0/0/77 | 0/77 | 0/77 |
| Pawitan  [7] | 159 | NA. Samples are classified into molecular subtypes. | 0/0/159 | 0/0/159 | 28/58/61/12 | 0/159 | 0/159 |

## Meta-analysis methods

The change in probe set intensity between ER+ and ER- tumors in each study is expressed as an effect size (*d*) which is a standardized index measuring the magnitude of a treatment or covariate effect. The unbiased effect size (*d’*), which is an effect size estimate corrected for sample size bias, was calculated as follows: (1) *d =* $\overline{X}_{ER+}-\overline{X}_{ER-}$ / $SD_{p}$, (2) *d’ = 3d / (4 (n-2) – 1)* where $\overline{X}_{ER+}$ is the mean of the logged intensity in the ER+ tumors, $\overline{X}_{ER-}$ is the mean logged intensity in ER- tumors, and *SD_p_* is the pooled standard deviation. The estimated variance of d’ ($\hat{\sigma}_{d}^{2}$) is given by $\hat{\sigma}_{d}^{2}=(n_{ER+}^{-1}+n_{ER-}^{-1})+d^{2}(2(n_{ER+}+n_{ER-}))^{-1}$. Unbiased effect sizes in every dataset were calculated for every probe set on the HG-U133A chip, and combined using a random effects model to obtain an estimate of the probe set’s overall mean effect size which was then divided by the standard error and expressed as a *Z* score. A *Z* score < 0 indicated a probe set with higher intensity in ER- tumors. A measure of the significance of this differential expression was calculated by converting the Z scores to *p*-values which were then adjusted for multiple testing using the Benjamini-Yekutieli (BY) correction [8].

## Weighted average ratio

The weighted average ratio (*WAR*) for each probe-set was calculated as follows:

$WAR=\sum_{i} n_{i}\frac{\overline{I}_{ER+}}{\overline{I}_{ER-}}$ / $\sum_{i} n_{i}$where *n_i_* is the number of tumors in the study *i*, $\overline{I}_{ER+}$ is the probe set’s mean un-logged intensity in ER+ tumors and $\overline{I}_{ER-}$ is its mean un-logged intensity in ER- tumors. In order to provide an accurate representation of those probe sets with higher intensities in ER- tumors, the reported WAR was calculated as follows.

*WAR* > 1, reported *WAR = WAR*

*WAR* < 1, reported *WAR = ­-*1/*WAR*

## Functional annotation analysis

Functional analysis of sets of selected genes was carried out using the gene-enrichment annotation analysis tools within DAVID version 2007 [9]*.* Categories analyzed included GO categories (Biological Process, Molecular Function, and Cellular Component), protein domain categories (InterPro Name, Superfamily Name, SMART Name), pathways databases (BBID, BioCarta, KEGG Pathways), functional categories (COG/KOG Ontology, Sp Pir Keywords, Up Seq Feature) and a disease category (Genetic Association Database).

## References:

1. Farmer P, Bonnefoi H, Becette V, Tubiana-Hulin M, Fumoleau P, et al. (2005) Identification of molecular apocrine breast tumours by microarray analysis. Oncogene 24: 4660-4671.

2. Sotiriou C, Wirapati P, Loi S, Harris A, Fox S, et al. (2006) Gene expression profiling in breast cancer: understanding the molecular basis of histologic grade to improve prognosis. J Natl Cancer Inst 98: 262-272.

3. Miller LD, Smeds J, George J, Vega VB, Vergara L, et al. (2005) An expression signature for p53 status in human breast cancer predicts mutation status, transcriptional effects, and patient survival. Proc Natl Acad Sci U S A 102: 13550-13555.

4. Minn AJ, Gupta GP, Siegel PM, Bos PD, Shu W, et al. (2005) Genes that mediate breast cancer metastasis to lung. Nature 436: 518-524.

5. Richardson AL, Wang ZC, De Nicolo A, Lu X, Brown M, et al. (2006) X chromosomal abnormalities in basal-like human breast cancer. Cancer Cell 9: 121-132.

6. Wang Y, Klijn JG, Zhang Y, Sieuwerts AM, Look MP, et al. (2005) Gene-expression profiles to predict distant metastasis of lymph-node-negative primary breast cancer. Lancet 365: 671-679.

7. Pawitan Y, Bjohle J, Amler L, Borg AL, Egyhazi S, et al. (2005) Gene expression profiling spares early breast cancer patients from adjuvant therapy: derived and validated in two population-based cohorts. Breast Cancer Res 7: R953-R964.

8. Benjamini Y, Yekutieli D (2001) The control of the false discovery rate in multiple testing under dependency. Ann Statist 29: 1165–1188.

9. Dennis G Jr., Sherman BT, Hosack DA, Yang J, Gao W, et al. (2003) DAVID: Database for Annotation, Visualization and Integrated Discovery. Genome Biology 4: R60.
