# Supplementary material for: Meta-Analysis and Gene Set Enrichment Relative to ER Status Reveal Elevated Activity of MYC and E2F in the “Basal” Breast Cancer Subgroup
Source: PLoS One. 2009 Mar 9;4(3):e4710. doi: 10.1371/journal.pone.0004710 (PMC2650420; doi:10.1371/journal.pone.0004710)
Supplement: Text S2 — Behavior of major biological categories enriched or depleted in ER− tumors in GSEA. Tables are sorted by increasing FDR. Gene sets linked with Estrogen (E2), MYC or E2F activity have been labeled with suffixes “U”, “D”, “B”, “Dir” and “M” referring to whether the genes in the set are Up-regulated or Down-regulated by the molecule in question, Binding Partners (or direct targets) of the molecule identified via ChIP, other Direct targets of the molecule identified by the use of cycloheximide, or whether they contain conserved Binding Motifs for the molecule in their promoter regions. (0.47 MB DOC) [file pone.0004710.s010.doc]

**Text S2. Behavior of major biological categories enriched or depleted in ER- tumors in the meta-analysis and/or GSEA.**

[A. Breast Cancer 2](#__RefHeading___Toc191298303)

[B. Cell Cycle 3](#__RefHeading___Toc191298304)

[C. Estrogen datasets 4](#__RefHeading___Toc191298305)

[D. MYC datasets 7](#__RefHeading___Toc191298306)

[E. E2F datasets 12](#__RefHeading___Toc191298307)

[References: 16](#__RefHeading___Toc191298308)

**Behavior of major biological categories enriched or depleted in ER- tumors in GSEA.**

The results from the second GSEA screen are indicated. Tables are sorted by increasing FDR. Gene sets linked with Estrogen (E_2_), MYC or E2F activity have been labeled with suffixes “U”, “D”, “B”, “Dir” and “M” referring to whether the genes in the set are **U**p-regulated or **D**own-regulated by the molecule in question, **B**inding Partners (or direct targets) of the molecule identified via ChIP, other **Dir**ect targets of the molecule identified by the use of cycloheximide, or whether they contain conserved Binding **M**otifs for the molecule in their promoter regions.

## A. Breast Cancer

**i) Gene sets enriched in ER- tumors**

| **Gene Set Name** | **Ref** | **System** | **Description** | **FDR** |
| --- | --- | --- | --- | --- |
| BRCA_ER_NEG | [1] | Breast tumors | Genes whose expression is consistently negatively correlated with estrogen receptor status in breast cancer | **<0.0001** |
| BRCA_PROGNOSIS_NEG | [1] | Breast tumors | Genes whose expression is consistently negatively correlated with breast cancer outcomes - higher expression is associated with metastasis and poor prognosis | **<0.0001** |
| VANTVEER_BREAST_OUTCOME_  GOOD_VS_POOR_DN | [1] | Breast tumors | Poor prognosis marker genes in Breast Cancer (part of NKI-70) from [1] | **<0.0001** |
| BRCA_BRCA1_POS | [1] | Breast tumors | Genes whose expression is consistently positively correlated with BRCA1 germline status in breast cancer - higher expression is associated with sporadic tumors | **<0.0001** |

**ii) Gene sets depleted in ER- tumors**

| **Gene Set Name** | **Ref** | **System** | **Description** | **FDR** |
| --- | --- | --- | --- | --- |
| BRCA_ER_POS | [1] | Breast tumors | Genes whose expression is consistently positively correlated with estrogen receptor status in breast cancer | **<0.0001** |
| MCA.ABBAS2005_SAGE_ERPOS_U | [2] | Breast tumors | Genes up in ER+ | **<0.0001** |
| BRCA_BRCA1_NEG | [1] | Breast tumors | Genes whose expression is consistently negatively correlated with brca1 germline status in breast cancer - higher expression is associated with BRCA1 tumors | **<0.0001** |
| BRCA_PROGNOSIS_POS | [1] | Breast tumors | Genes whose expression is consistently positively correlated with breast cancer outcomes - higher expression is associated with good prognosis | **<0.0001** |
| VANTVEER_BREAST_OUTCOME_  GOOD_VS_POOR_UP | [1] | Breast tumors | Good prognosis marker genes in Breast Cancer (part of NKI-70) | **<0.0001** |

## B. Cell Cycle

**i) Gene sets enriched in ER- tumors**

| **Gene Set Name** | **Ref** | **System** | **Description** | **FDR** |
| --- | --- | --- | --- | --- |
| CELL_CYCLE_KEGG | [3] | Generic | Curated in GenMapp | **<0.0001** |
| CELL_CYCLE |  | Gene Ontology | The progression of biochemical and morphological events that occur during nuclear or cellular replication. | **<0.0001** |
| DNA_REPLICATION_REACTOME | [3] | Generic | Curated in GenMapp | **<0.0001** |
| CIS_XPC_UP | [4] | Human fibroblasts | Increased expression in XPC-defective fibroblasts, compared to normal fibroblasts, following treatment with cisplatin (XPC is a DNA damage recognition protein). | **0.0076** |
| G1_TO_S_CELL_CYCLE_REACTOME | [3] | Generic | Curated in GenMapp | **0.087** |
| G2PATHWAY |  | Generic | Activated Cdc2-cyclin B kinase regulates the G2/M transition; DNA damage stimulates the DNA-PK/ATM/ATR kinases, which inactivate Cdc2. Curated in BioCarta | **0.0098** |
| ATMPATHWAY |  | Generic | The tumor-suppressing protein kinase ATM responds to radiation-induced DNA damage by blocking cell-cycle progression and activating DNA repair. Curated in Biocarta. | **0.035** |
| APOPTOSIS | [3] | Generic | Curated in GenMapp | **0.043** |

**ii) Gene sets depleted in ER- tumors**

| **Gene Set Name** | **Ref** | **System** | **Description** | **FDR** |
| --- | --- | --- | --- | --- |
| RNA_TRANSCRIPTION_REACTOME | [3] | Generic | Curated in GenMapp | 0.38 |

## C. Estrogen datasets

**i) Gene sets enriched in ER- tumors**

| **Gene Set Name** | **Ref** | **System** | **Description** | **FDR** |
| --- | --- | --- | --- | --- |
| MCA.Creighton.Cluster B Genes.  E2_Early.Sustained_U | [5] | MCF-7, T-47D and BT-474  Up to 24hrs of E2-stimulation. | E2_U  Induced within 4 hours, with sustained induction through 24hrs. | **0.0047** |
| MCA.Musgrove_E2_U | [6]** | Anti-estrogen arrested MCF-7.  6 hours after E_2_-induction. | E2_U | **0.015** |
| MCA.Creighton.Cluster E Genes.  E2_Early.Short_D | [5] | MCF-7, T-47D and BT-474.  Up to 24h of E2-stimulation | E2_D  Repressed within 4 hours, then return to baseline. | 0.070 |
| MCA.Oh_E2_U | [7] | MCF-7  Up to 24hrs of E2-stimulation. | E2_U  Genes induced by E_2_ at 2, 4, 8 and 24 hours | 0.17 |
| MCA.Creighton.Cluster A Genes.  E2_Early.Short_U | [5] | MCF-7, T-47D and BT-474  Up to 24h of E2-stimulation. | E2_U  Induced within 4 hours, then return to baseline. | 0.25 |
| MCA.Carroll_E2_3hr_U | [8] ** | MCF-7  3 hrs of E_2_ stimulation. | E2_U | 0.30 |
| MCA.Charpentier_E2_3hr_SAGE_U | [9] | MCF-7  10 hrs of E_2_ stimulation. | E2_U | 0.35 |
| MCA.Cheng_ER_B | [10] | MCF-7  ChIP | ER_B | 0.73 |

**ii)** Gene sets depleted in ER- tumors

| **Gene Set Name** | **Ref** | **System** | **Description** | **FDR** |
| --- | --- | --- | --- | --- |
| MCA.Oh_E2_D | [7] | MCF-7  Up to 24hrs of E2-stimulation. | E2_D  Genes repressed by E_2_ at 2, 4, 8 and 24 hours | **<0.0001** |
| FRASOR_ER_UP | [11] | MCF-7 | SERM up-regulated signature | **0.0004** |
| MCA.Carroll_E2_3hr_U and ER_50kb_B | [8] ** | MCF-7  3hrs of E_2_ stimulation.  ChIP identification of ER binding sites within 50 kb of transcription start site (TSS). | E2_U and ER_B | **0.0013** |
| MCA.Soulez_E2_6hr_B | [12] | MCF-7  6hrs of E_2_ stimulation.  Cyclohexamide | E2_Dir | **0.0022** |
| MCA.Carroll_E2_6hr_U and ER_50kb_B | [8] ** | MCF-7  6hrs of E_2_ stimulation.  ChIP identification of ER binding sites within 50 kb of TSS. | E2_U and ER_B | **0.0032** |
| MCA.Musgrove_E2_D | [6]** | Anti-estrogen arrested MCF-7.  6 hours after E_2_-induction. | E2_D | **0.0052** |
| MCA.Carroll_E2_6hr_D and ER_50kb_B | [8] ** | MCF-7  6hrs of E_2_ stimulation.  ChIP identification of ER binding sites within 50 kb of TSS. | E2_D and ER_B | **0.0051** |
| MCA.Laganiere_ER_B | [13] | MCF-7 (45mins)  ChIP | ER_B | **0.011** |
| FRASOR_ER_DN | [11] | MCF-7 | SERM down-regulated signature | **0.014** |
| MCA.Carroll_E2_3hr_D and ER_50kb_B | [8] ** | MCF-7  3hrs of E_2_ stimulation.  ChIP identification of ER binding sites within 50 kb of TSS. | E2_D and ER_B | **0.021** |
| MCA.Carroll_ER_Promoter-Proximal_B | [8] | MCF-7  ChIP identification of ER-binding sites within proximal promoter region (-800 bp to +200 bp). | ER_B | **0.021** |
| MCA.Carroll_ER_50kb_B | [8] | MCF-7  ChIP identification of ER binding sites within 50 kb of TSS. | ER_B | **0.023** |
| MCA.Carroll_E2_3hr_D | [8] ** | MCF-7  3hrs of E_2_ stimulation. | E2_D | **0.028** |
| MCA.Kwon_ER_B | [14] | MCF-7  1hr of E_2_-stimulation  ChIP-DSL identification of ER binding sites within proximal promoter region. | ER_B | 0.052 |
| MCA.Creighton.Cluster F Genes. E2_Early.Sustained_D | [5] | MCF-7, T-47D and BT-474  Up to 24hrs of E2-stimulation. | E2_D  Repressed within 4 hrs, with sustained repression through to 24hrs. | 0.053 |
| MCA.Charpentier_E2_3hr_SAGE_D | [9] | MCF-7  3hrs of E_2_ stimulation. | E2_D | 0.11 |
| BREAST_CANCER_  ESTROGEN_SIGNALING |  | Generic | Genes preferentially expressed in breast cancers, especially those involved in ER-dependent signal transduction. Contributed by GEArray. | 0.32 |
| STOSSI_ER_UP | [15] | U2OS human osteosarcoma cells | Genes up-regulated by E_2_ through ERalpha and ERbeta in U2OS cells | 0.38 |

** Data derived from experiment referenced, and re-analyzed as per “Materials and Methods”

## D. MYC datasets

**i) Enrichment plot of E_2_-induced and MYC-induced genes in MCF-7 cells (MCA.Musgrove_E2_U_and_Myc_U).**

A positive correlation indicates a gene within the set with higher expression in ER- tumors.

**
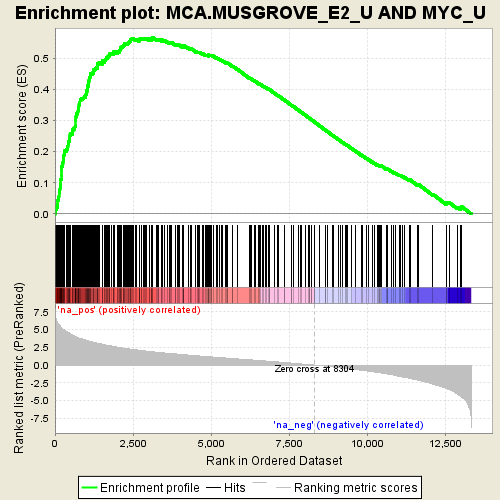
**

**ii) MYC-related gene sets enriched in ER- tumors**

| **Gene Set Name** | **Ref** | **Experimental System** | **Description** | **FDR** |
| --- | --- | --- | --- | --- |
| MCA.Musgrove_E2_U and Myc_U | [6]** | Anti-estrogen arrested MCF-7.  6 hours after E_2_-induction, or  6 hours after MYC- induction. | E2_U and MYC_U | **<0.0001** |
| MCA.Musgrove_Myc_U | [6]** | Anti-estrogen arrested MCF-7.  6 hours after MYC- induction. | MYC_U | **<0.0001** |
| MCA.Zeller_Myc_U and Myc_B | [16] | Human B lymphoid tumor.  ChIP coupled with pair-end ditag sequencing analysis (ChIP-PET) | MYC_U and MYC_B | **<0.0001** |
| MCA.Bild_Myc.LIMMA_U | [17] ** | HMEC | MYC_U | **<0.0001** |
| YU_CMYC_UP | [18] | Non-transgenic murine model for B-cell lymphoma. | MYC_U | **<0.0001** |
| SCHUMACHER_MYC_UP | [19] | Human P-493-6 B lymphocytes | MYC_U | **0.0002** |
| MCA.Li_Myc_Structure.Function_U | [20] | P-493-6 | MYC_U | **0.0002** |
| MCA.Lawlor_Myc_Early_2hr.or.4hr_U | [21] | Reversible transgenic murine model of MYC activation in tumorigenesis | MYC_U | **0.0003** |
| BASSO_REGULATORY_HUBS | [22] | Human B cells | Genes which comprise the top 1% of highly interconnected genes (major hubs) that account for most of the interactions in the reconstructed regulatory networks from expression profiles in human B cells. MYC is a major hub. | **0.0016** |
| MCA.O.Connell_Myc_U | [23] | TGR-1 rat fibroblasts | MYC_U | **0.0028** |
| MYC_TARGETS | [24] | Literature. Most genes validated by ChIP | MYC_U+D_B | **0.0046** |
| COLLER_MYC_UP | [25] | 293T (transformed fetal renal cells) | MYC_U | **0.0072** |
| MENSSEN_MYC_UP | [26] | Primary human umbilical vein endothelial cells (HUVEC) | MYC_U | **0.0074** |
| MCA.Mao_Myc_U and Myc_B | [27] | HL-60.  ChIP | MYC_U and MYC_B | **0.011** |
| V$MYC_Q2 | [28] | NA | MYC_M | **0.018** |
| FERNANDEZ_MYC_TARGETS | [29] | U-937 (monoblastic leukemia),  HL-60 (myeloid leukemia), P-493-6 cell lines  ChIP | MYC_B | **0.019** |
| ZELLER_MYC_UP | [24] | Literature. | MYC_U in >3 papers | **0.023** |
| MCA.Watson_Myc_U | [30] | TGR-1 | MYC_U | **0.031** |
| MCA.Guo_Myc_U | [31] | TGR-1 | MYC_U | **0.040** |
| MCA.Mao_MYC_B and E2F1_B or.E2F4_B | [27] | HL-60 | MYC_B and E2F1_B or E2F4_B | 0.071 |
| V$MYCMAX_01 | [28] | NA | MYC_M | 0.09 |
| MCA.Lawlor_Myc_Early_U | [21] | Reversible transgenic murine model of MYC activation in tumorigenesis | MYC_U | 0.11 |
| MCA.LAWLOR_MYC_SUSTAINED_U | [21] | Reversible transgenic murine model of MYC activation in tumorigenesis | MYC_U | 0.14 |
| V$MYCMAX_02 | [28] | NA | MYC_M | 0.21 |
| MCA.Mao_Myc_B | [27] | HL-60 | MYC_B | 0.24 |
| CACGTG_V$MYC_Q2 | [28] | NA | MYC_M | 0.28 |
| V$MYCMAX_03 | [28] | NA | MYC_M | 0.37 |
| MCA.GUO_MYC_D | [31] | TGR-1 | MYC_D | 0.41 |
| MCA.LAWLOR_MYC_REGRESSION | [21] | Reversible transgenic murine model of MYC activation in tumorigenesis |  | 0.43 |
| V$MYCMAX_B | [28] | NA | MYC_M | 0.50 |
| MCA.Bild_Myc.LIMMA_D | [17] ** | HMEC | MYC_D | 0.60 |
| MCA.ZELLER_MYC_D AND MYC_B | [16] | Human B lymphoid tumor.  ChIP coupled with pair-end ditag sequencing analysis (ChIP-PET) | MYC_D and MYC_B | 0.97 |
| MCA.PERINI_N-MYC_B | [32] | SK-N-BE, a human neuroblastoma cell line expressing  N-MYC but not c-MYC | N-MYC _B | 0.98 |

** Data derived from experiment referenced, and re-analyzed as per “Materials and Methods”

**iii) MYC-related g**ene sets depleted in ER- tumors

| **Gene Set Name** | **Ref** | **Experimental System** | **Description** | **FDR** |
| --- | --- | --- | --- | --- |
| MCA.Musgrove_ Myc_D | [6]** | Anti-estrogen arrested MCF-7.  6 hours after MYC-induction. | MYC_D | **<0.0001** |
| MCA.Musgrove_E2_D and Myc_D | [6]** | Anti-estrogen arrested MCF-7.  6 hours after E_2_-induction, or  6 hours after MYC-induction. | E2_D and MYC_D | **<0.0001** |
| MCA.Musgrove_E2_U and Myc_D | [6]** | Anti-estrogen arrested MCF-7.  6 hours after E_2_-induction, or  6 hours after MYC-induction. | E2_U and MYC_D | **0.0006** |
| MCA.Watson_Myc_D | [30] | TGR-1 rat fibroblasts | MYC_D | 0.56 |
| MCA.O.Connell_Myc_D | [23] | TGR-1 rat fibroblasts | MYC_D | 0.64 |
| MCA.Lawlor_Myc_Early_2hr.or.4hr_D | [21] | Reversible transgenic murine model of MYC activation in tumorigenesis | MYC_D | 0.73 |
| MCA.Cawley_Myc.Chr21.22_B | [33] | Jurkat cells.  ChIP to identify MYC binding sited on Chr21 and 22. | MYC_B | 0.96 |

** Data derived from experiment referenced, and re-analyzed as per “Materials and Methods”

## E. E2F datasets

**i) Enrichment plot of direct targets of E2F4 during G_0_ phase in mouse embryonic fibroblasts (MCA.Baliciunate_E2F4.G0_B) [34]**.

**
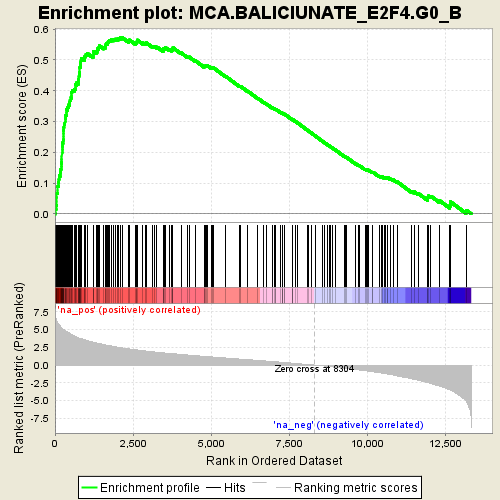
**

**ii) E2F-related g**ene sets enriched in ER- tumors

| **Gene Set Name** | **Reference** | **Experimental System** | **Description** | **FDR** |
| --- | --- | --- | --- | --- |
| MCA.Baliciunate_p130.G1_B | [34] | Mouse embryonic fibroblasts (MEFs)  (G_1_ phase)  ChIP | P130_B | **<0.0001** |
| MCA.Baliciunate_E2F4.G0_B | [34] | MEF (G_0_ phase)  ChIP | E2F4_B | **<0.0001** |
| MCA.Baliciunate_p107.G1_B | [34] | MEF (G_1_ phase)  ChIP | P107_B | **<0.0001** |
| MCA.BLACK.2005.TOP.100.E2F1-INDUCED GENES.SUPPLTABLE2 | [35] | MEFs | E2F1_U | **<0.0001** |
| VERNELL_PRB_CLSTR1 | [36] | U2OS | E2F_U  Up-regulated by E2Fs 1,2 or 3 and down-regulated by pRB and p16 | <**0.0001** |
| MCA.Baliciunate_p130.G0_B | [34] | MEF  (G_0_ phase)  ChIP | P130_B | **<0.0001** |
| MCA.Baliciunate_E2F4.G1_B | [34] | MEF  (G_1_ phase)  ChIP | E2F4_B | **<0.0001** |
| MCA.BLACK.2005.TOP.100.E2F3-INDUCED GENES.SUPPLTABLE3 | [35] | MEFs | E2F3_U | **<0.0001** |
| REN_E2F1_TARGETS | [37] | WI-38 Primary human fibroblasts  ChIP | E2F1_B and E2F4_B | **<0.0001** |
| SGCGSSAAA_V$E2F1DP2_01 | [28] | NA | E2F1_M and TFDP2_M | **<0.0001** |
| V$E2F1_Q6 | [28] | NA | E2F1 _M | **<0.0001** |
| V$E2F4DP2_01 | [28] | NA | E2F4_M +TFDP2_M | **<0.0001** |
| V$E2F1_Q6_01 | [28] | NA | E2F1 _M | **<0.0001** |
| V$E2F1DP2_01 | [28] | NA | E2F1_M and TFDP2_M | **0.0001** |
| V$E2F1DP1_01 | [28] | NA | E2F1_M and TFDP1_M | **0.0001** |
| V$E2F_02 | [28] | NA | E2F_M | **0.0002** |
| V$E2F4DP1_01 | [28] | NA | E2F4+TFDP1_M | **0.0002** |
| V$E2F1_Q3 | [28] | NA | E2F1_M | **0.0002** |
| MCA.REN.E2F4_B.NOT.E2F1_B | [37] | WI-38  ChIP | E2F4_B not E2F1_B | **0.0007** |
| V$E2F_Q6 | [28] | NA | E2F_M | **0.0028** |
| V$E2F_01 | [28] | NA | E2F_M | **0.0037** |
| V$E2F_Q4 | [28] | NA | E2F_M | **0.0038** |
| V$E2F_Q4_01 | [28] | NA | E2F_M | **0.0046** |
| V$E2F_03 | [28] | NA | E2F_M | **0.0046** |
| V$E2F_Q3 | [28] | NA | E2F_M | **0.0050** |
| V$E2F1_Q4_01 | [28] | NA | E2F1_M | **0.0069** |
| V$E2F_Q3_01 | [28] | NA | E2F_M | **0.0088** |
| V$E2F_Q6_01 | [28] | NA | E2F_M | **0.0096** |
| MCA.XU_TABLE S4.TOP RANKED E2F PROMOTERS_COMBINED E2F1, E2F4, OR E2F6 IN ALL 5 CELL TYPES | [38] | MCF-7, MCF-10A, HeLa, GM06690 and Ntera2 cells | E2F1_B or E2F4_B or E2F6_B | **0.012** |
| V$E2F1DP1RB_01 | [28] |  | E2F1+TFDP1+RB_M | **0.014** |
| MCA.Weinmann_E2F4_B | [39] | HeLa  CpG island arrays | E2F4_B | **0.032** |
| V$E2F1_Q4 | [28] |  | E2F1_M | 0.15 |
| MCA.Bild_E2F3.LIMMA_U | [17] | HMEC | E2F3_U | 0.17 |
| MCA.Bild_E2F3.LIMMA_D | [17] | HMEC | E2F3_D | 0.22 |
| V$E2F1_Q3_01 | [28] | NA | E2F_M | 0.25 |
| V$E2F_Q2 | [28] | NA | E2F_M | 0.25 |
| MCA.XU.TOP250.E2F1_B.IN.MCF7.CELLS | [38] | MCF-7 | E2F1_B | 0.28 |
| LEE_E2F1_UP | [40] | Hepatocellular carcinomas (HCC) | Up-regulated in hepatoma tissue of E2f1 transgenic mice. | 0.46 |
| STANELLE_E2F1_UP | [41] | Osteosarcoma | E2F1_U | 0.49 |
| MCA.JIN_E2F1_B | [42];  personal communication | MCF-7 Promoter tiling array (Chr11 to Chr 23) | E2F1_B | 0.52 |

**iii) E2F-related gene sets** depleted in ER- tumors

| **Gene Set Name** | **Ref** | **System** | **Description** | **FDR** |
| --- | --- | --- | --- | --- |
| VERNELL_PRB_CLSTR2 | [36] | U2OS cells | E2F_D  Down-regulated by E2Fs 1,2 or 3 and up-regulated by pRB and p16 | **0.038** |
| MCA.OBERLEY_E2F6_B | [43] | Human 293 cells | E2F6_B | 0.75 |

## References:

1. van 't Veer LJ, Dai H, van de Vijver MJ, He YD, Hart AA, et al. (2002) Gene expression profiling predicts clinical outcome of breast cancer. Nature 415: 530-536.

2. Abba MC, Hu Y, Sun H, Drake JA, Gaddis S, et al. (2005) Gene expression signature of estrogen receptor alpha status in breast cancer. BMC Genomics 6: 37.

3. Dahlquist KD, Salomonis N, Vranizan K, Lawlor SC, Conklin BR (2002) GenMAPP, a new tool for viewing and analyzing microarray data on biological pathways. Nature Genetics 31: 19-20.

4. Wang G, Chuang L, Zhang X, Colton S, Dombkowski A, et al. (2004) The initiative role of XPC protein in cisplatin DNA damaging treatment-mediated cell cycle regulation. Nucl Acids Res 32: 2231-2240.

5. Creighton CJ, Cordero KE, Larios JM, Miller RS, Johnson MD, et al. (2006) Genes regulated by estrogen in breast tumor cells in vitro are similarly regulated in vivo in tumor xenografts and human breast tumors. Genome Biol 7: R28.

6. Musgrove EA, Sergio CM, Loi S, Inman CK, Anderson LR, et al. (2008) Identification of Functional Networks of Estrogen- and c-Myc-Responsive Genes and Their Relationship to Response to Tamoxifen Therapy in Breast Cancer. PLoS ONE 3: e2987.

7. Oh DS, Troester MA, Usary J, Hu Z, He X, et al. (2006) Estrogen-Regulated Genes Predict Survival in Hormone Receptor-Positive Breast Cancers. J Clin Oncol 24: 1656-1664.

8. Carroll JS, Meyer CA, Song J, Li W, Geistlinger TR, et al. (2006) Genome-wide analysis of estrogen receptor binding sites. Nature Genetics 38: 1289-1297.

9. Charpentier AH, Bednarek AK, Daniel RL, Hawkins KA, Laflin KJ, et al. (2000) Effects of estrogen on global gene expression: identification of novel targets of estrogen action. Cancer Res 60: 5977-5983.

10. Cheng ASL, Jin VX, Fan M, Smith LT, Liyanarachchi S, et al. (2006) Combinatorial Analysis of Transcription Factor Partners Reveals Recruitment of c-MYC to Estrogen Receptor-a Responsive Promoters. Molecular Cell 21: 393-404.

11. Frasor J, Stossi F, Danes JM, Komm B, Lyttle CR, et al. (2004) Selective estrogen receptor modulators: discrimination of agonistic versus antagonistic activities by gene expression profiling in breast cancer cells. Cancer Reseach 64: 1522-1533.

12. Soulez M, Parker MG (2001) Identification of novel oestrogen receptor target genes in human ZR75-1 breast cancer cells by expression profiling. Journal of Molecular Endocrinology 27: 259-274.

13. Laganiere J, Deblois G, Lefebvre C, Bataille AR, Robert F, et al. (2005) Location analysis of estrogen receptor {alpha} target promoters reveals that FOXA1 defines a domain of the estrogen response. PNAS 102: 11651-11656.

14. Kwon Y-S, Garcia-Bassets I, Hutt KR, Cheng CS, Jin M, et al. (2007) Sensitive ChIP-DSL technology reveals an extensive estrogen receptor {alpha}-binding program on human gene promoters. PNAS 104: 4852-4857.

15. Stossi F, Barnett DH, Frasor J, Komm B, Lyttle CR, et al. (2004) Transcriptional profiling of estrogen-regulated gene expression via estrogen receptor (ER) alpha or ERbeta in human osteosarcoma cells: distinct and common target genes for these receptors. Endocrinology 145: 3473-3486.

16. Zeller KI, Zhao X, Lee CWH, Chiu KP, Yao F, et al. (2006) Global mapping of c-Myc binding sites and target gene networks in human B cells. PNAS 103: 17834-17839.

17. Bild AH, Yao G, Chang JT, Wang Q, Potti A, et al. (2006) Oncogenic pathway signatures in human cancers as a guide to targeted therapies. Nature 439: 353-357.

18. Yu D, Cozma D, Park A, Thomas-Tikhonenko A (2005) Functional validation of genes implicated in lymphomagenesis: an in vivo selection assay using a Myc-induced B-cell tumor. Ann N Y Acad Sci 1059: 145-159.

19. Schuhmacher M, Kohlhuber F, Holzel M, Kaiser C, Burtscher H, et al. (2001) The transcriptional program of a human B cell line in response to Myc. Nucleic Acids Res 29: 397-406.

20. Li F, Wang Y, Zeller KI, Potter JJ, Wonsey DR, et al. (2005) Myc stimulates nuclearly encoded mitochondrial genes and mitochondrial biogenesis. Mol Cell Biol 25: 6225-6234.

21. Lawlor ER, Soucek L, Brown-Swigart L, Shchors K, Bialucha CU, et al. (2006) Reversible Kinetic Analysis of Myc Targets In vivo Provides Novel Insights into Myc-Mediated Tumorigenesis. Cancer Res 66: 4591-4601.

22. Basso K, Margolin AA, Stolovitzky G, Klein U, Dalla-Favera R, et al. (2005) Reverse engineering of regulatory networks in human B cells. Nature Genetics 37: 382-390.

23. O'Connell BC, Cheung AF, Simkevich CP, Tam W, Ren X, et al. (2003) A large scale genetic analysis of c-Myc-regulated gene expression patterns. J Biol Chem 278: 12563-12573.

24. Zeller KI, Jegga AG, Aronow BJ, O'Donnell KA, Dang CV (2003) An integrated database of genes responsive to the Myc oncogenic transcription factor: identification of direct genomic targets. Genome Biol 4: R69.

25. Coller HA, Grandori C, Tamayo P, Colbert T, Lander ES, et al. (2000) Expression analysis with oligonucleotide microarrays reveals that MYC regulates genes involved in growth, cell cycle, signaling, and adhesion. Proc Natl Acad Sci U S A 97: 3260-3265.

26. Menssen A, Hermeking H (2002) Characterization of the c-MYC-regulated transcriptome by SAGE: identification and analysis of c-MYC target genes. Proc Natl Acad Sci U S A 99: 6274-6279.

27. Mao DYL, Watson JD, Yan PS, Barsyte-Lovejoy D, Khosravi F, et al. (2003) Analysis of Myc Bound Loci Identified by CpG Island Arrays Shows that Max Is Essential for Myc-Dependent Repression. Current Biology 13: 882-886.

28. Xie X, Lu J, Kulbokas EJ, Golub TR, Mootha V, et al. (2005) Systematic discovery of regulatory motifs in human promoters and 3[prime] UTRs by comparison of several mammals. Nature 434: 338-345.

29. Fernandez PC, Frank SR, Wang L, Schroeder M, Liu S, et al. (2003) Genomic targets of the human c-Myc protein. Genes Dev 17: 1115-1129.

30. Watson JD, Oster SK, Shago M, Khosravi F, Penn LZ (2002) Identifying genes regulated in a Myc-dependent manner. J Biol Chem 277: 36921-36930.

31. Guo QM, Malek RL, Kim S, Chiao C, He M, et al. (2000) Identification of c-myc responsive genes using rat cDNA microarray. Cancer Res 60: 5922-5928.

32. Perini G, Diolaiti D, Porro A, Della Valle G (2005) In vivo transcriptional regulation of N-Myc target genes is controlled by E-box methylation. PNAS 102: 12117-12122.

33. Cawley S, Bekiranov S, Ng HH, Kapranov P, Sekinger EA, et al. (2004) Unbiased mapping of transcription factor binding sites along human chromosomes 21 and 22 points to widespread regulation of noncoding RNAs. Cell 116: 499-509.

34. Balciunaite E, Spektor A, Lents NH, Cam H, te Riele H, et al. (2005) Pocket Protein Complexes Are Recruited to Distinct Targets in Quiescent and Proliferating Cells. Mol Cell Biol 25: 8166-8178.

35. Black EP, Hallstrom T, Dressman HK, West M, Nevins JR (2005) Distinctions in the specificity of E2F function revealed by gene expression signatures. Proceedings of the National Academy of Sciences 102: 15948-15953.

36. Vernell R, Helin K, Muller H (2003) Identification of Target Genes of the p16INK4A-pRB-E2F Pathway. J Biol Chem 278: 46124-46137.

37. Ren B, Cam H, Takahashi Y, Volkert T, Terragni J, et al. (2002) E2F integrates cell cycle progression with DNA repair, replication, and G2/M checkpoints. Genes Dev 16: 245-256.

38. Xu X, Bieda M, Jin VX, Rabinovich A, Oberley MJ, et al. (2007) A comprehensive ChIP chip analysis of E2F1, E2F4, and E2F6 in normal and tumor cells reveals interchangeable roles of E2F family members. Genome Res 17: 1550-1561.

39. Weinmann AS, Yan PS, Oberley MJ, Huang TH-M, Farnham PJ (2002) Isolating human transcription factor targets by coupling chromatin immunoprecipitation and CpG island microarray analysis. Genes Dev 16: 235-244.

40. Lee J-S, Chu I-S, Mikaelyan A, Calvisi DF, Heo J, et al. (2004) Application of comparative functional genomics to identify best-fit mouse models to study human cancer. Nature Genetics 36: 1306-1311.

41. Stanelle J, Stiewe T, Theseling CC, Peter M, Pützer BM (2002) Gene expression changes in response to E2F1 activation. Nucleic Acids Res 30: 1859-1867.

42. Jin VX, Rabinovich A, Squazzo SL, Green R, Farnham PJ (2006) A computational genomics approach to identify cis-regulatory modules from chromatin immunoprecipitation microarray data--A case study using E2F1. Genome Res 16: 1585-1595.

43. Oberley MJ, Inman DR, Farnham PJ (2003) E2F6 Negatively Regulates BRCA1 in Human Cancer Cells without Methylation of Histone H3 on Lysine 9. J Biol Chem 278: 42466-42476.
