# Supplementary material for: Meta-Analysis and Gene Set Enrichment Relative to ER Status Reveal Elevated Activity of MYC and E2F in the “Basal” Breast Cancer Subgroup
Source: PLoS One. 2009 Mar 9;4(3):e4710. doi: 10.1371/journal.pone.0004710 (PMC2650420; doi:10.1371/journal.pone.0004710)
Supplement: Text S3 — Behavior of probe sets for MYC, E2F and associated genes in the meta-analysis. (0.09 MB DOC) [file pone.0004710.s011.doc]

## Text S3 - Behavior of probe sets for MYC, E2F and associated genes in the meta-analysis.

## A. Meta-analysis results of Myc transcript and interacting proteins

**Higher expression in ER- tumors**

| Probe.Set.ID | Gene.Title | Gene Symbol | Chromosomal Location | Un-adjusted P | Adjusted P |
| --- | --- | --- | --- | --- | --- |
| 202431_s_at | v-myc myelocytomatosis viral oncogene homolog (avian) | MYC | chr8q24.12-q24.13 | 0.00028 | **0.024** |
| 203964_at | N-myc (and STAT) interactor | NMI | chr2p24.3-q21.3 | 0.0017 | 0.10 |
| 215491_at | v-myc myelocytomatosis viral oncogene homolog 1, lung carcinoma derived (avian) | MYCL1 | chr1p34.2 | 0.0063 | 0.28 |
| 214058_at | v-myc myelocytomatosis viral oncogene homolog 1, lung carcinoma derived (avian) | MYCL1 | chr1p34.2 | 0.0070 | 0.31 |
| 213188_s_at | MYC induced nuclear antigen | MINA | chr3q11.2 | 0.0083 | 0.35 |
| 213189_at | MYC induced nuclear antigen | MINA | chr3q11.2 | 0.0098 | 0.39 |
| 200632_s_at | N-myc downstream regulated gene 1 | NDRG1 | chr8q24.3 | 0.013 | 0.49 |
| 207028_at | v-myc myelocytomatosis viral related oncogene, neuroblastoma derived (avian) opposite strand | MYCNOS | chr2p24.1 | 0.067 | 1.00 |
| 201959_s_at | MYC binding protein 2 | MYCBP2 | chr13q22 | 0.090 | 1.00 |
| 209757_s_at | v-myc myelocytomatosis viral related oncogene, neuroblastoma derived (avian) | MYCN | chr2p24.1 | 0.13 | 1.00 |
| 212945_s_at | MAX gene associated | MGA | chr15q14 | 0.18 | 1.00 |
| 201960_s_at | MYC binding protein 2 | MYCBP2 | chr13q22 | 0.19 | 1.00 |
| 216188_at | v-myc myelocytomatosis viral related oncogene, neuroblastoma derived (avian) opposite strand | MYCNOS | chr2p24.1 | 0.22 | 1.00 |
| 220471_s_at | myc target 1 | MYCT1 | chr6q25.2 | 0.34 | 1.00 |
| 214787_at | c-myc promoter binding protein | MYCPBP | chr15q22.31 | 0.34 | 1.00 |
| 211377_x_at | v-myc myelocytomatosis viral related oncogene, neuroblastoma derived (avian) | MYCN | chr2p24.1 | 0.36 | 1.00 |

**Lower expression in ER- tumors**

| Probe.Set.ID | Gene.Title | Gene. Symbol | Chromosomal. Location | Un-adjusted P | Adjusted P |
| --- | --- | --- | --- | --- | --- |
| 203359_s_at | c-myc binding protein | MYCBP | chr1p33-p32.2 | 3.54E-05 | **0.0047** |
| 203361_s_at | c-myc binding protein | MYCBP | chr1p33-p32.2 | 0.00011 | 0.011 |
| 209331_s_at | MYC associated factor X | MAX | chr14q23 | 0.00012 | 0.013 |
| 209332_s_at | MYC associated factor X | MAX | chr14q23 | 0.00068 | 0.049 |
| 207824_s_at | MYC-associated zinc finger protein (purine-binding transcription factor) | MAZ | chr16p11.2 | 0.011 | 0.43 |
| 203360_s_at | c-myc binding protein | MYCBP | chr1p33-p32.2 | 0.025 | 0.79 |
| 214108_at | MYC associated factor X | MAX | chr14q23 | 0.16 | 1.00 |
| 210734_x_at | MYC associated factor X | MAX | chr14q23 | 0.19 | 1.00 |
| 208403_x_at | MYC associated factor X | MAX | chr14q23 | 0.26 | 1.00 |
| 212064_x_at | MYC-associated zinc finger protein (purine-binding transcription factor) | MAZ | chr16p11.2 | 0.30 | 1.00 |
| 209756_s_at | v-myc myelocytomatosis viral related oncogene, neuroblastoma derived (avian) | MYCN | chr2p24.1 | 0.42 | 1.00 |

## B. Meta-analysis results for probe sets of individual E2Fs and interacting proteins

**Higher expression in ER- tumors**

| **Gene**  **Symbol** | **Gene Title** | **Chromosomal**  **Location** | **Probe set ID** | **Un-adjusted P** | **BY-adjusted P** |
| --- | --- | --- | --- | --- | --- |
| E2F3 | E2F transcription factor 3 | chr6p22 | 203693_s_at | 8.60E-14 | **6.54E-10** |
| E2F3 | E2F transcription factor 3 | chr6p22 | 203692_s_at | 1.29E-10 | **2.12E-07** |
| TFDP2 | transcription factor Dp-2 (E2F dimerization partner 2) | chr3q23 | 203588_s_at | 1.49E-05 | **0.0023** |
| TFDP1 | transcription factor Dp-1 | chr13q34 | 212330_at | 6.08E-05 | **0.0073** |
| E2F8 | E2F transcription factor 8 | chr11p15.1 | 219990_at | 0.00043 | **0.034** |
| E2F1 | E2F transcription factor 1 | chr20q11.2 | 204947_at | 0.0029 | 0.15 |
| E2F4 | E2F transcription factor 4, p107/p130-binding | chr16q21-q22 | 202248_at | 0.0051 | 0.24 |
| E2F1 | E2F transcription factor 1 | chr20q11.2 | 2028_s_at | 0.014 | 0.51 |
| RBL1 | retinoblastoma-like 1 (p107) | chr20q11.2 | 205296_at | 0.022 | 0.72 |
| TFDP1 | transcription factor Dp-1 | chr13q34 | 204147_s_at | 0.024 | 0.76 |
| E2F5 | E2F transcription factor 5, p130-binding | chr8q21.2 | 221586_s_at | 0.030 | 0.88 |
| TFDP2 | transcription factor Dp-2 (E2F dimerization partner 2) | chr3q23 | 203589_s_at | 0.039 | 1.00 |
| E2F5 | E2F transcription factor 5, p130-binding | chr8q21.2 | 222051_s_at | 0.10 | 1.00 |
| TFDP3 | transcription factor Dp family, member 3 | chrXq26.2 | 207385_at | 0.10 | 1.00 |
| E2F2 | E2F transcription factor 2 | chr1p36 | 207042_at | 0.15 | 1.00 |
| RB1 | retinoblastoma 1 (including osteosarcoma) | chr13q14.2 | 211540_s_at | 0.46 | 1.00 |

**Lower expression in ER- tumors:**

| **Gene**  **Symbol** | **Gene Title** | **Chromosomal**  **Location** | **Probe set ID** | **Un-adjusted P** | **BY-adjusted P** |
| --- | --- | --- | --- | --- | --- |
| RBL2 | retinoblastoma-like 2 (p130) | chr16q12.2 | 212331_at | 4.05E-05 | **0.0053** |
| RB1 | retinoblastoma 1 (including osteosarcoma) | chr13q14.2 | 203132_at | 0.01 | 0.40 |
| RBL2 | retinoblastoma-like 2 (p130) | chr16q12.2 | 212332_at | 0.093 | 1.00 |
| E2F4 | E2F transcription factor 4, p107/p130-binding | chr16q21-q22 | 38707_r_at | 0.37 | 1.00 |
| E2F6 | E2F transcription factor 6 | chr2p25.1 | 203957_at | 0.44 | 1.00 |
